# Low-grade glioneuronal tumors with *FGFR2* fusion resolve into a single epigenetic group corresponding to ‘Polymorphous low-grade neuroepithelial tumor of the young’

**DOI:** 10.1007/s00401-021-02352-w

**Published:** 2021-07-28

**Authors:** Rohit Gupta, Calixto-Hope G. Lucas, Jasper Wu, Jairo Barreto, Kathan Shah, Iraide Bernal Simon, Sandro Casavilca-Zambrano, Carole Brathwaite, Holly Zhou, Dario Caccamo, Ahmed Gilani, Bette K. Kleinschmidt-DeMasters, Julieann C. Lee, Arie Perry, Jennifer L. Clarke, Susan M. Chang, Mitchel S. Berger, David A. Solomon

**Affiliations:** 1 Department of Pathology, University of California, San Francisco, 513 Parnassus Ave, Health Sciences West 451, San Francisco, CA 94143 USA; 2 Department of Pathology, University Hospital Donostia, San Sebastián, Spain; 3 Department of Pathology, National Institute of Neoplastic Diseases, Lima, Peru; 4 Department of Pathology, Nicklaus Children’s Hospital, Miami, FL USA; 5 Department of Pathology, University of Utah School of Medicine, Salt Lake City, UT USA; 6 Department of Pathology, Sutter Medical Center, Sacramento, CA USA; 7 Department of Pathology, University of Colorado, Aurora, CO USA; 8 Department of Neurological Surgery, University of California, San Francisco, CA USA; 9 Division of Neuro-Oncology, Department of Neurological Surgery, University of California, San Francisco, CA USA; 10 Department of Neurology, University of California, San Francisco, CA USA

Low-grade neuroepithelial tumors (LGNET) are a diverse group of neoplasms occurring most commonly in children and young adults, often associated with epilepsy and favorable clinical outcomes. They are composed of a spectrum of tumor entities with divergent clinicopathologic features including ganglioglioma, pilocytic astrocytoma, dysembryoplastic neuroepithelial tumor (DNT), rosette-forming glioneuronal tumor (RGNT), extraventricular neurocytoma (EVN), multinodular and vacuolating neuronal tumor (MVNT), polymorphous low-grade neuroepithelial tumor of the young (PLTNY), myxoid glioneuronal tumor (MGNT), diffuse leptomeningeal glioneuronal tumor (DLGNT), and papillary glioneuronal tumor (PGNT). However, histologically distinguishing between these different LGNET subtypes can be challenging, and molecular profiling is now recognized as critical for accurate classification. While some LGNET subtypes are defined by unique genetic alterations (*e.g. PRKCA* fusion in PGNT [4], *PDGFRA* p.K385L/I dinucleotide mutation in MGNT [9]) that can be used for definitive subtyping, other alterations such as *BRAF* mutation or fusion are nonspecific and can be seen in ganglioglioma, pilocytic astrocytoma, MVNT, and DLGNT [3, 10–12, 14]. *FGFR1* is another promiscuous oncogene in LGNET with kinase domain tandem duplication, gene fusions (most often with *TACC1* as the fusion partner), or hotspot missense mutations at one of two codons within the tyrosine kinase domain (p.N546 or p.K656) recurrently found in pilocytic astrocytoma, DNT, RGNT, and EVN [8, 12–17, 20]. Thus, additional ancillary methodologies such as DNA methylation profiling may be necessary for accurate classification of LGNET with either *BRAF* or *FGFR1* alterations.

Fusions involving the related *FGFR2* oncogene have recently been described as one of the characteristic genetic alterations in the newly recognized tumor entity PLNTY, an epileptogenic neoplasm predominantly occurring in the cerebral hemispheres of children and young adults with oligodendroglioma‑like components, abundant calcification, and aberrant CD34 expression [5]. However, rare cases of histologically-defined ganglioglioma, MVNT, DNT, oligodendroglioma, and unclassifiable low-grade glioneuronal tumors have also been reported with *FGFR2* fusions [6, 10, 11, 14]. To improve classification for such *FGFR2*-fused tumors, we performed targeted next-generation sequencing and genome-wide DNA methylation profiling on a cohort of 9 patients with LGNET harboring *FGFR2* fusions with a diverse range of histologic diagnoses. Three patients had been previously included in our investigations on the genomic landscape of ganglioglioma and MVNT [10, 11]. The cohort consisted of 6 males and 3 females with median age of 11 years (range 6–38 years), all presenting with chronic seizures (Fig. 1a). Imaging revealed solid and cystic lesions within the cerebral hemispheres (Fig. 1b; Supplementary Fig. 1 [Online Resource 1]). Most patients had gross total resection associated with resolution of seizures and freedom from recurrence during the period of clinical follow-up without adjuvant therapy (Supplementary Table 1 [Online Resource 2]; Supplementary Fig. 2 [Online Resource 1]). Histologically, these were low-grade infiltrative gliomas composed of tumor cells with predominantly round oligodendrocyte-like nuclei (Fig. 1c; Supplementary Fig. 3 [Online Resource 1]; Supplementary Table 2 [Online Resource 2]). Calcifications, often extensive, were present in most but not all tumors. A subset of tumors demonstrated a dysmorphic ganglion cell component and eosinophilic granular bodies (Fig. 1d). Hemosiderin-laden macrophages, indicative of prior intra-tumoral hemorrhage, were also a common finding. One tumor demonstrated vacuolation in both the stroma and ganglion cell component resembling MVNT. Rosenthal fibers, necrosis, and microvascular proliferation were not encountered. Mitotic activity was inconspicuous, and Ki67 labeling was less than 2% in all examined tumors. Neurofilament and synaptophysin staining revealed entrapped axonal processes in the background of all tumors, and additionally highlighted the dysmorphic ganglion cell component in a subset (Supplementary Table 3 [Online Resource 2]). Immunohistochemistry for CD34 demonstrated diffuse strong labeling of tumor cells characteristic of PLNTY in 6 of 9 tumors, and showed only scattered ramified cells more characteristic of ganglioglioma in the other 3 tumors (Fig. 1e). The original histopathologic diagnosis was PLNTY (*n* = 5), ganglioglioma (*n* = 2), mixed MVNT/ganglioglioma (*n* = 1), and unclassifiable LGNET (*n* = 1). Four tumors demonstrated *FGFR2* fusion with *INA* as the partner, one tumor each demonstrated fusion with *KIAA1598*, *ACTR1A*, and *OPTN*, and two tumors demonstrated complex *FGFR2* rearrangements with uncertain fusion partner based on the targeted DNA sequencing analysis (Supplementary Table 4 [Online Resource 2]). The *FGFR2* fusion was the solitary pathogenic alteration identified in all tumors, with an absence of accompanying alterations involving *IDH1/2*, histone H3 genes, *BRAF*, *NF1*, *PRKCA*, *FGFR1*, *PIK3CA*, *PIK3R1*, *PTEN*, *CDKN2A*, *TP53*, *TERT* (including promoter region), *ATRX*, *CIC*, *FUBP1*, *MYB*, and *MYBL1* [7]. The quantity of chromosomal copy number aberrations was variable, but no tumors harbored whole arm co-deletion of chromosomes 1p and 19q, nor were there focal amplifications or homozygous deletions in any tumors (Supplementary Table 5 [Online Resource 2]).

**Fig. 1:**
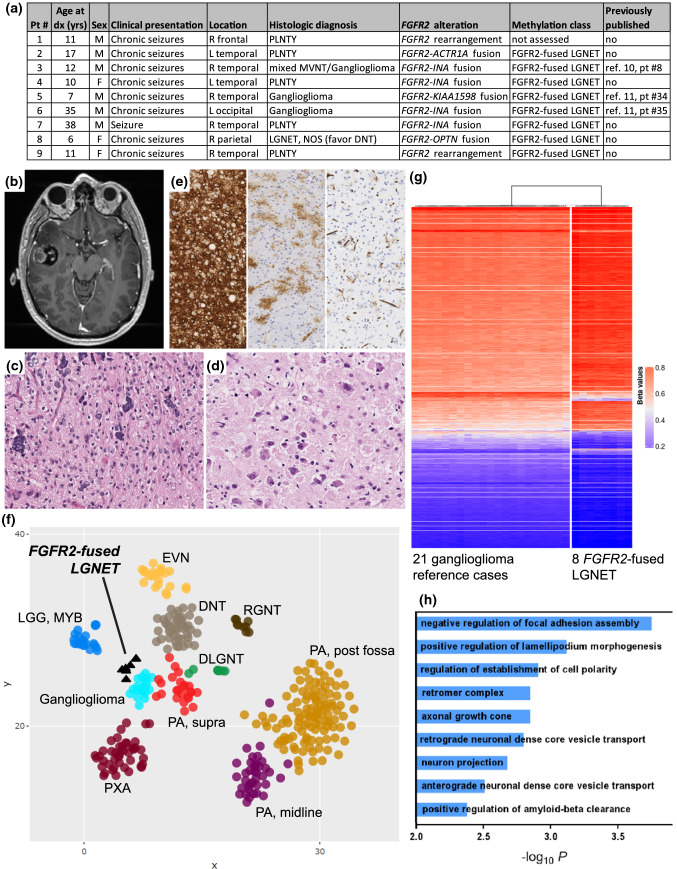
Clinicopathologic features and epigenomic profiling of low-grade neuroepithelial tumors harboring *FGFR2* gene fusions. **a** Summary table of the 9 patients with *FGFR2*-fused LGNET. **b** MR imaging from patient #7 demonstrating a solid and cystic lesion within the right temporal lobe of the brain. **c** Histology from patient #1 showing characteristic features of PLNTY including round oligodendrocyte-like tumor cells with extensive calcifications. **d** Histology from patient #5 showing numerous dysmorphic ganglion cells and eosinophilic granular bodies without calcifications. **e** Immunochemical staining for CD34 protein showing variable staining patterns in the *FGFR2*-fused LGNET, including diffuse strong staining of tumor cells (left, patient #3), abundant ramified cells (middle, patient #6), and minimal extravascular positivity (right, patient #8). **f** tSNE dimensionality reduction plot of genome-wide DNA methylation profiles from 8 *FGFR2*-fused LGNET alongside 346 reference CNS tumors spanning 10 LGNET entities. **g** Unsupervised hierarchical clustering of DNA methylation data showing segregation of the 8 *FGFR2*-fused LGNET from a reference cohort of 21 gangliogliomas. **h** Differential methylation-based Gene Ontology analysis for *FGFR2*-fused LGNET compared to ganglioglioma, represented in a bar plot of − log_10_ P values for the most differentially methylated gene networks

Genome-wide DNA methylation profiling was performed on 8 of the tumors using Infinium EPIC 850k Beadchips (Illumina) following the manufacturer’s recommended protocols (see Supplementary Methods [Online Resource 3]). tSNE clustering of the DNA methylation data alongside reference cohorts of CNS tumors revealed that the *FGFR2*-fused LGNET formed a single epigenetic group that was distinct from all methylation classes in the current version of the DKFZ classifier v11b4 (Fig. 1f; Supplementary Fig. 4 [Online Resource 1]; Supplementary Tables 6–7 [Online Resource 2]) [2]. Unsupervised hierarchical clustering successfully segregated the 8 *FGFR2*-fused LGNET from a reference cohort of 21 gangliogliomas (Fig. 1g). Gene Ontology analysis of the most differentially methylated gene regions between *FGFR2*-fused LGNET and ganglioglioma revealed gene networks involved in cell polarity, lamellipodium morphogenesis, and neuronal functions including growth cone projection and core vesicle transport (Fig. 1h; Supplementary Tables 8–10 [Online Resource 2]). tSNE dimensionality reduction of the DNA methylation data alongside 3 histologically-defined cases of PLNTY with *FGFR2* fusion from Huse et al. [5] demonstrated close clustering, indicating low-grade glioneuronal tumors with *FGFR2* fusion resolve into a single epigenetic group (Supplementary Fig. 5 [Online Resource 1]). Additionally, tSNE dimensionality reduction and unsupervised hierarchical clustering of 3 histologically-defined cases of PLNTY with *BRAF* p.V600E mutation from Huse et al. [5] demonstrated overall epigenetic similarity to *FGFR2*-fused LGNET but resolved into a separate methylation subgroup by both methodologies, suggesting that PLNTY may be composed of at least two distinct epigenetic subgroups—those with *FGFR2* fusion and those with *BRAF* p.V600E mutation (Supplementary Fig. 5 and 6 [Online Resource 1]).

Here, we demonstrate LGNET with *FGFR2* fusions exhibit a spectrum of histologic features, but share an epigenetic signature distinct from all of the reference LGNET methylation classes in the current version 11b4 of the DKFZ classifier. Although most *FGFR2*-fused LGNET belonging to this unique methylation class have histologic features aligning with PLNTY, a subset is devoid of calcifications, have CD34 positivity limited to scattered ramified cells, and contain a prominent ganglion cell component, thereby complicating their differentiation from ganglioglioma based on microscopic features alone. Furthermore, while *BRAF* and *FGFR1* alterations are promiscuous and recurrently present across various LGNET types, *FGFR2* fusions among LGNET appear to be quite specific to this distinct epigenetic subgroup of glioneuronal tumors. Notably, similar *FGFR2* gene fusions are frequent in intrahepatic cholangiocarcinoma for which clinical trials have shown promising efficacy of small molecule tyrosine kinase inhibitors such as erdafitinib [1, 19]. It remains unclear why *FGFR2* fusions are selected for in PLNTY and intrahepatic cholangiocarcinoma, whereas *FGFR1* alterations are selected for in pilocytic astrocytoma, DNT, RGNT, and EVN, but is likely to reflect differences in response to the ligand-binding specificity for the various fibroblast growth factors [18]. Future studies are required to define the full clinicopathologic spectrum of *FGFR2*-fused LGNET and the potential efficacy of genomically tailored therapy for affected patients.

## Supplementary Information

Below is the link to the electronic supplementary material.

**Online Resource 1.** Supplementary Figures 1-6. (PDF 46158 kb)

**Online Resource 2.** Supplementary Tables 1-10. (XLSX 980 kb)

**Online Resource 3.** Supplementary Methods. (PDF 401 kb)

## Data Availability

Scanned image files of H&E and CD34 stained sections from the 9 tumors in this cohort are available for downloading and viewing at the following link: https://figshare.com/projects/Low-grade_neuroepithelial_tumors_with_FGFR2_fusion/113418. DNA methylation array data files from this study are available from the Gene Expression Omnibus (GEO) repository under accession number GSE172081 (https://www.ncbi.nlm.nih.gov/geo/). Structural variant and copy number data are available in the electronic supplementary material. Raw sequencing data files are available upon request.
