# Supplementary material for: Low-grade glioneuronal tumors with FGFR2 fusion resolve into a single epigenetic group corresponding to ‘Polymorphous low-grade neuroepithelial tumor of the young’
Source: Acta Neuropathol. 2021 Jul 28;142(3):595–9. doi: 10.1007/s00401-021-02352-w (PMC8357689; doi:10.1007/s00401-021-02352-w)
Supplement: Supplementary file 1 — Online Resource 1. Supplementary Figures 1-6. (PDF 46158 kb) [file 401_2021_2352_MOESM1_ESM.pdf]

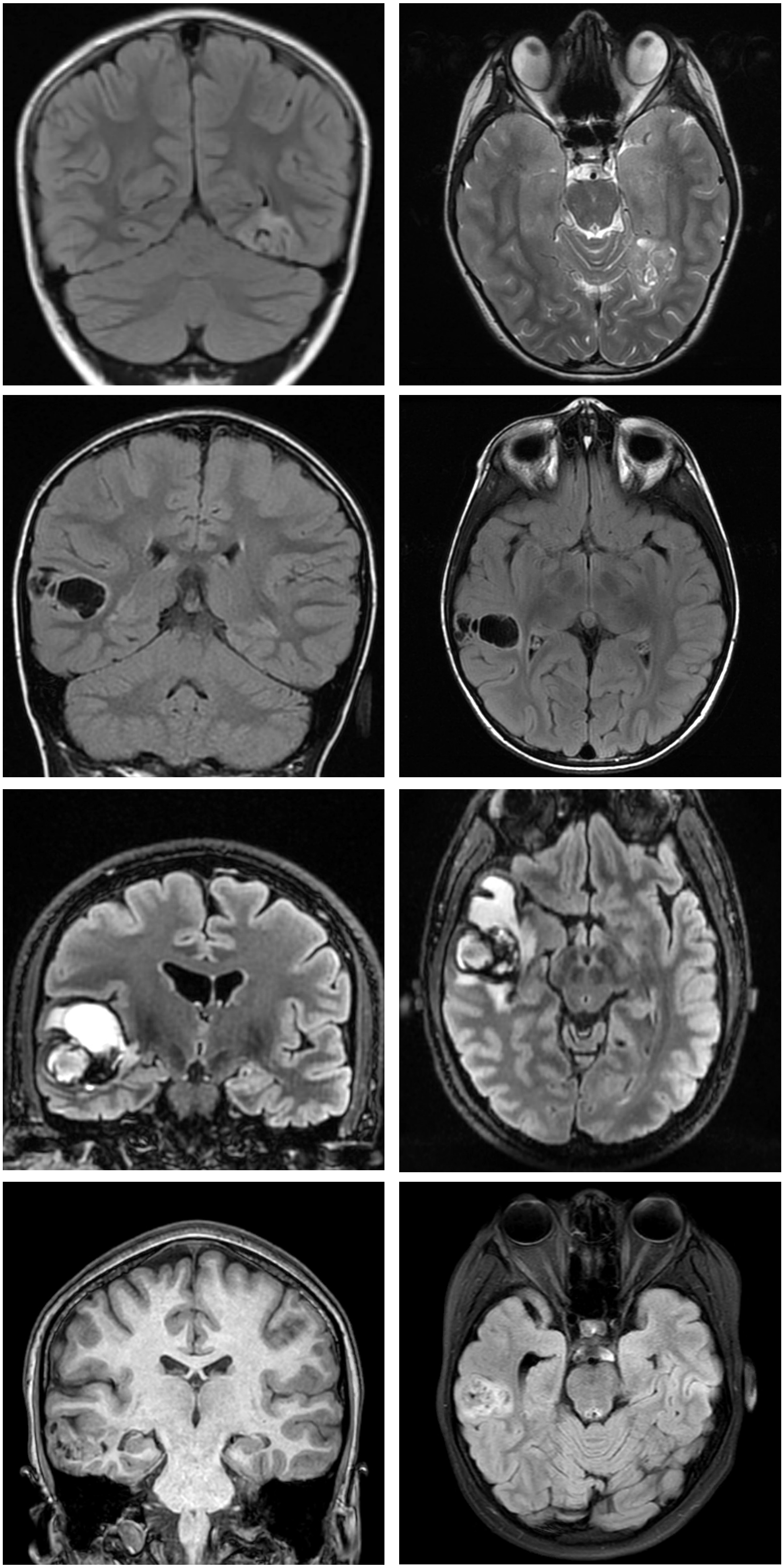


**Supplementary Figure 1.** Imaging features of low-grade neuroepithelial tumors with *FGFR2* fusion.

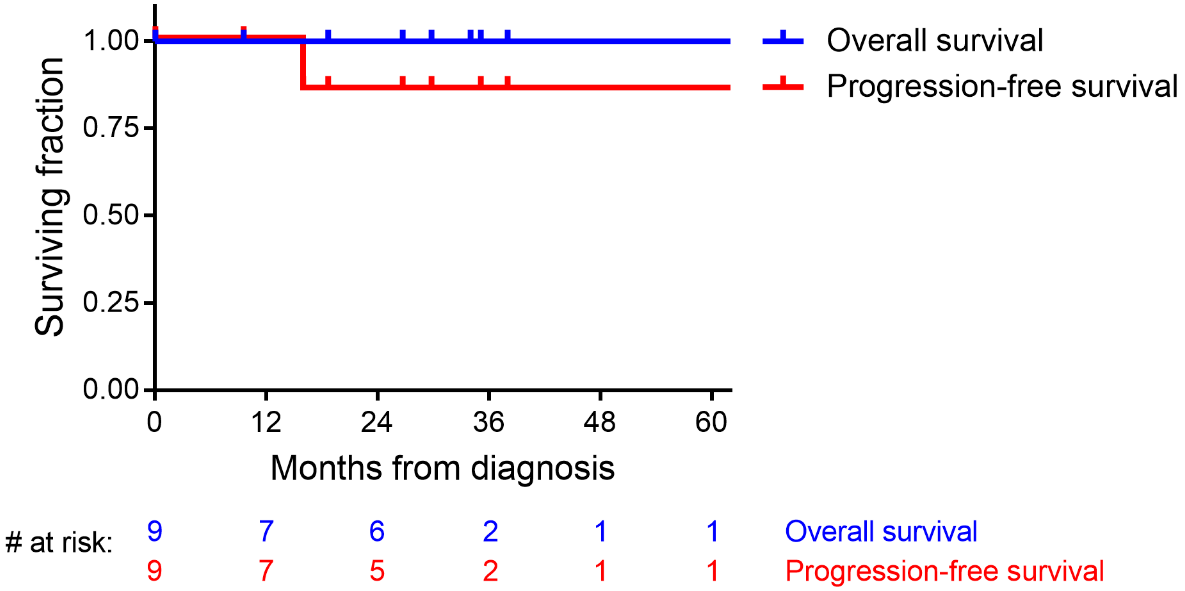


**Supplementary Figure 2.** Clinical outcomes for the 9 patients with low-grade neuroepithelial tumors harboring *FGFR2* fusions.

Patient #1, 11 y/o M, *FGFR2* rearrangement, institutional histologic diagnosis = PLNTY

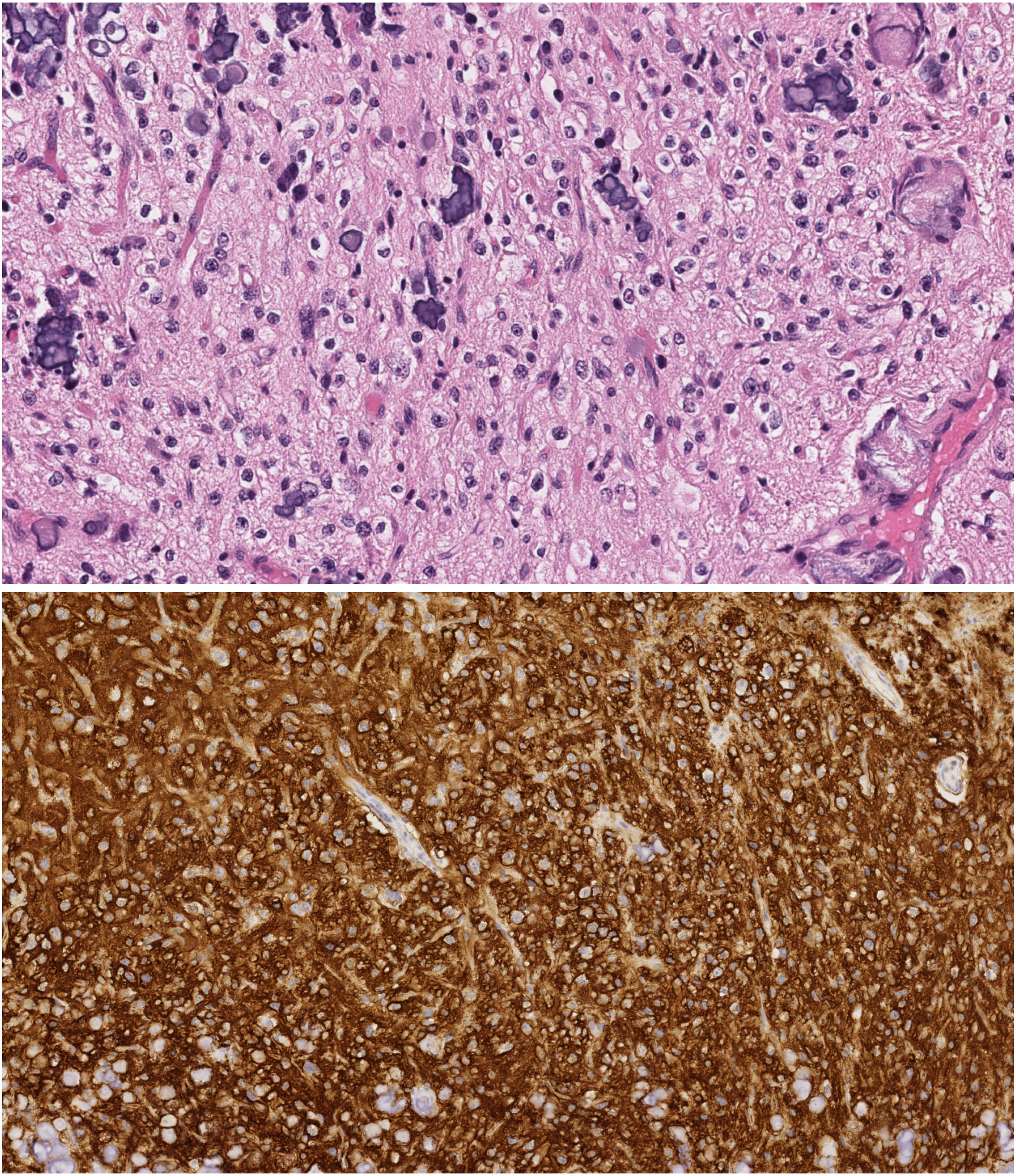

**Supplementary Figure 3.** Histologic features of low-grade neuroepithelial tumors with *FGFR2* fusion. Shown are an H&E stain at 40x mangification and a CD34 immunostain at 20x magnification for each of the nine tumors.

Patient #2, 17 y/o M, *FGFR2-ACTR1A* fusion, institutional histologic diagnosis = PLNTY

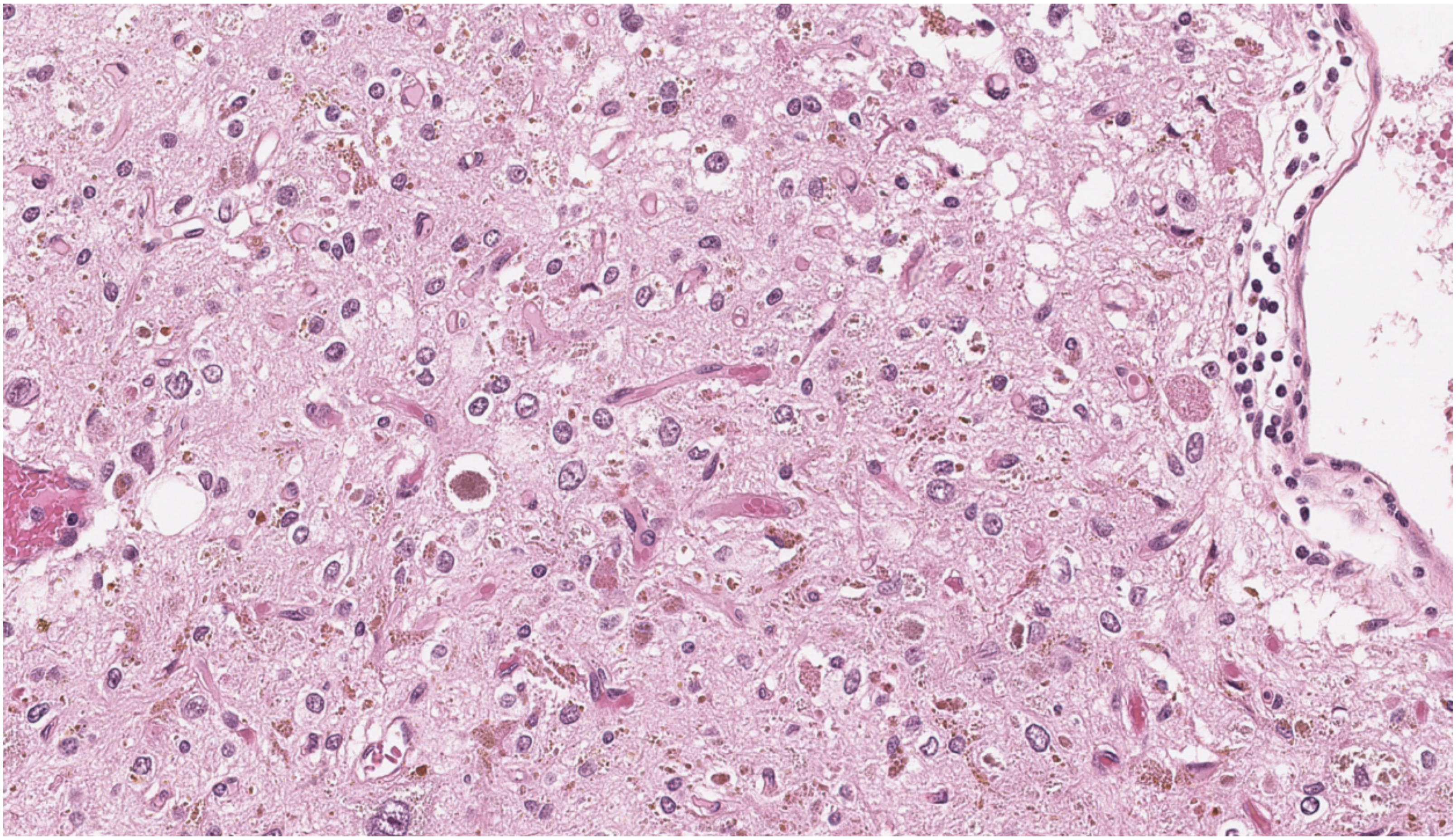

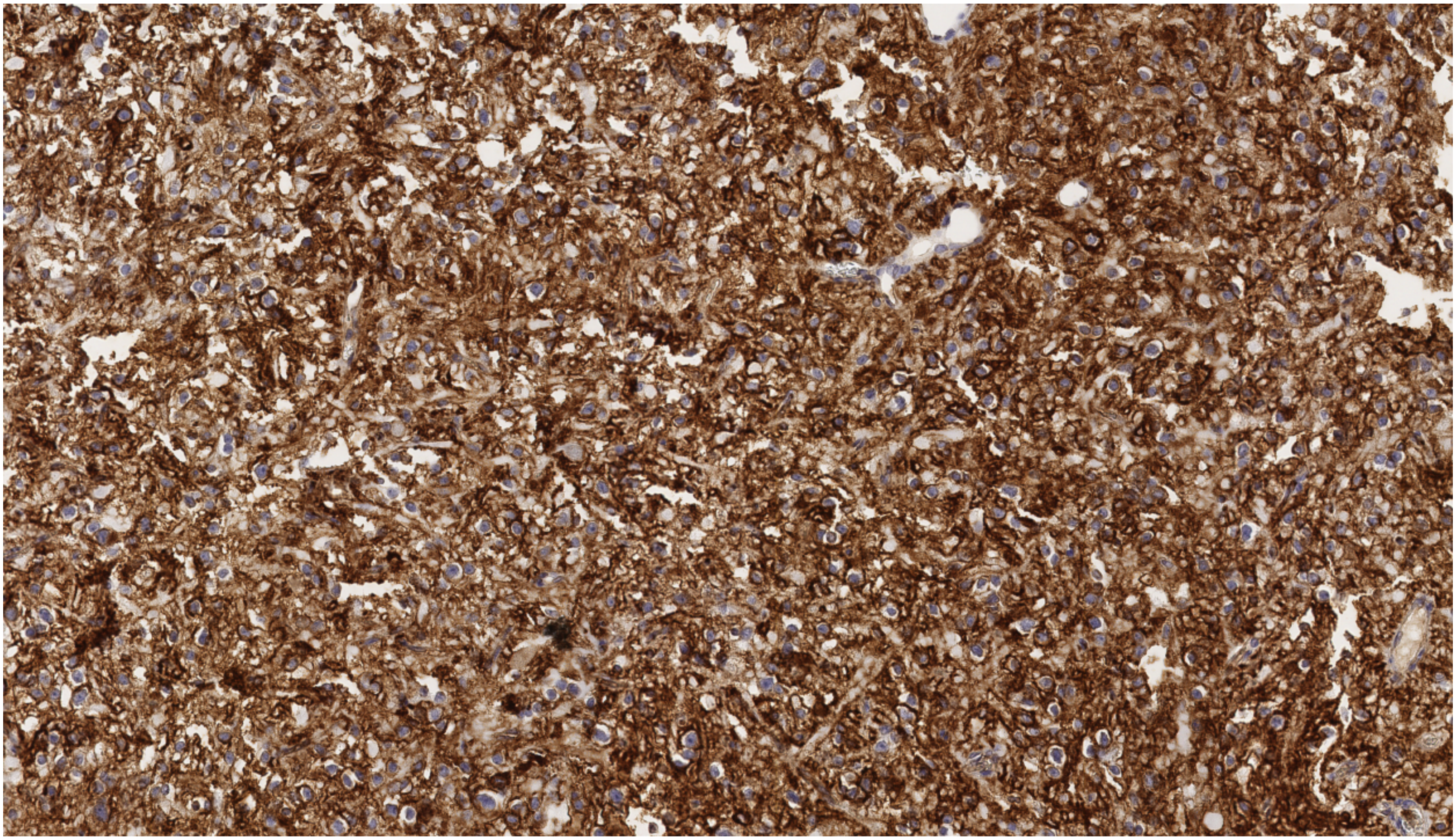

Patient #3, 12 y/o M, *FGFR2-INA* fusion, institutional histologic diagnosis = mixed MVNT/ganglioglioma

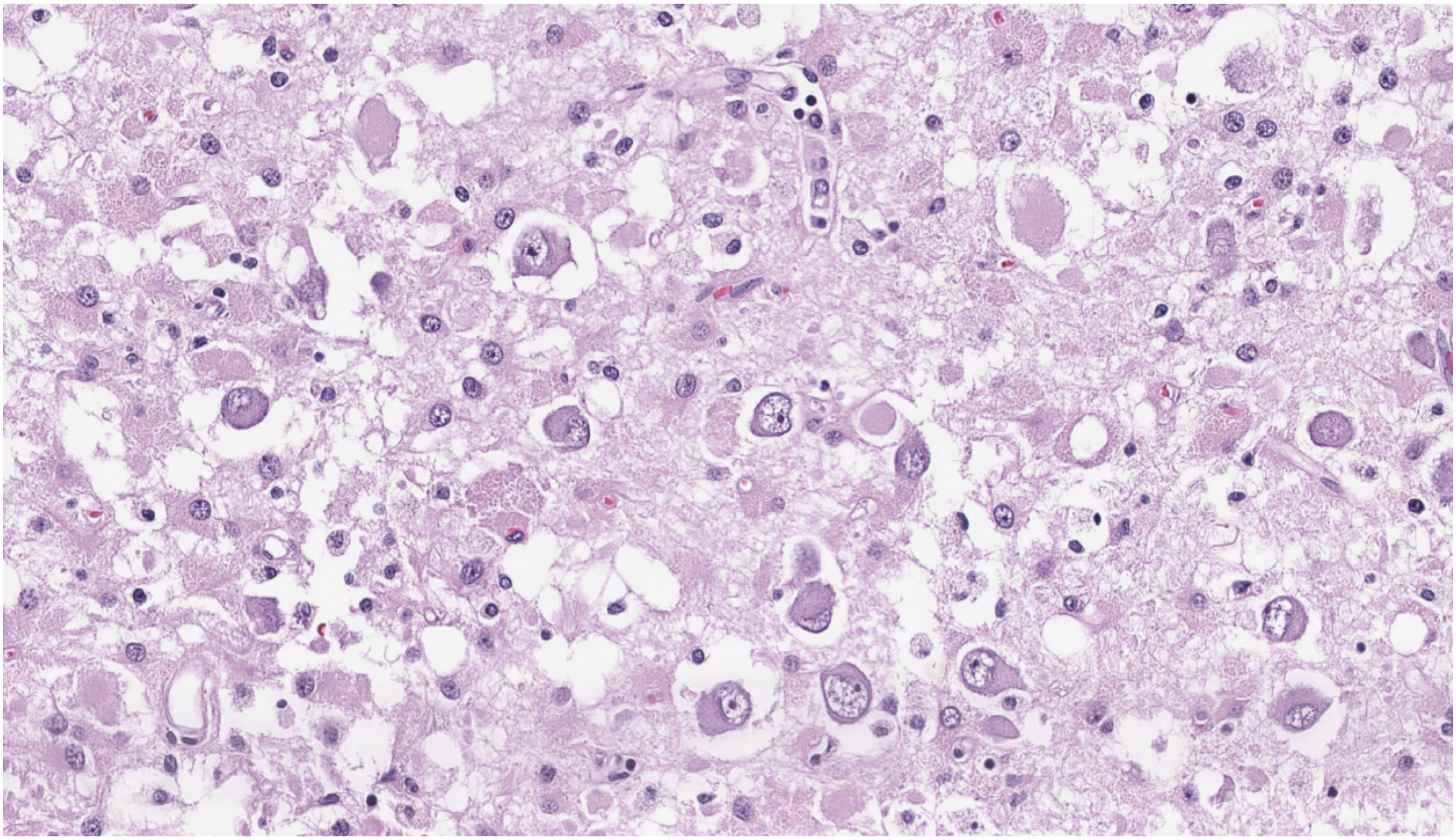

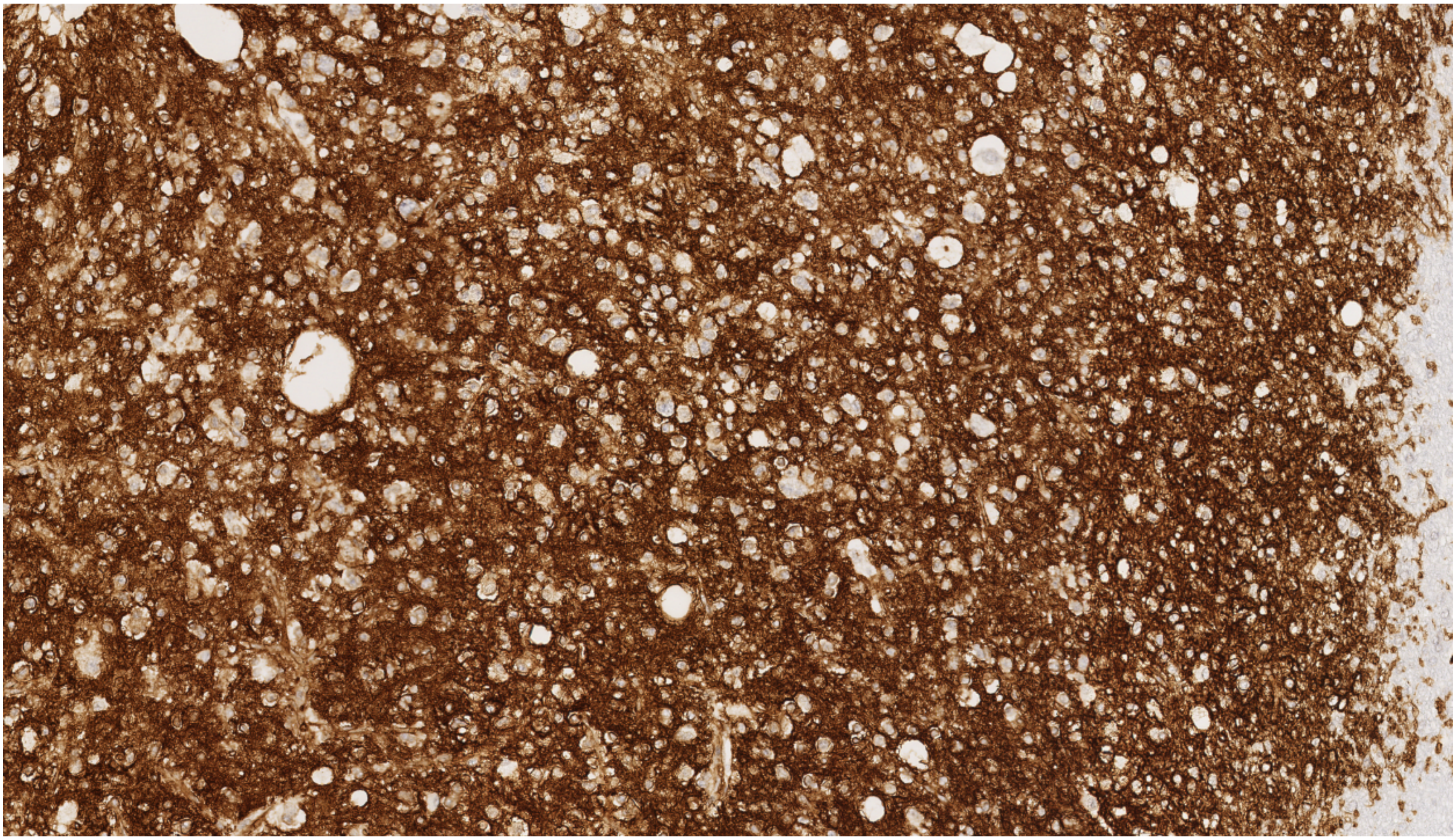

Patient #4, 10 y/o F, *FGFR2-INA* fusion, institutional histologic diagnosis = PLNTY

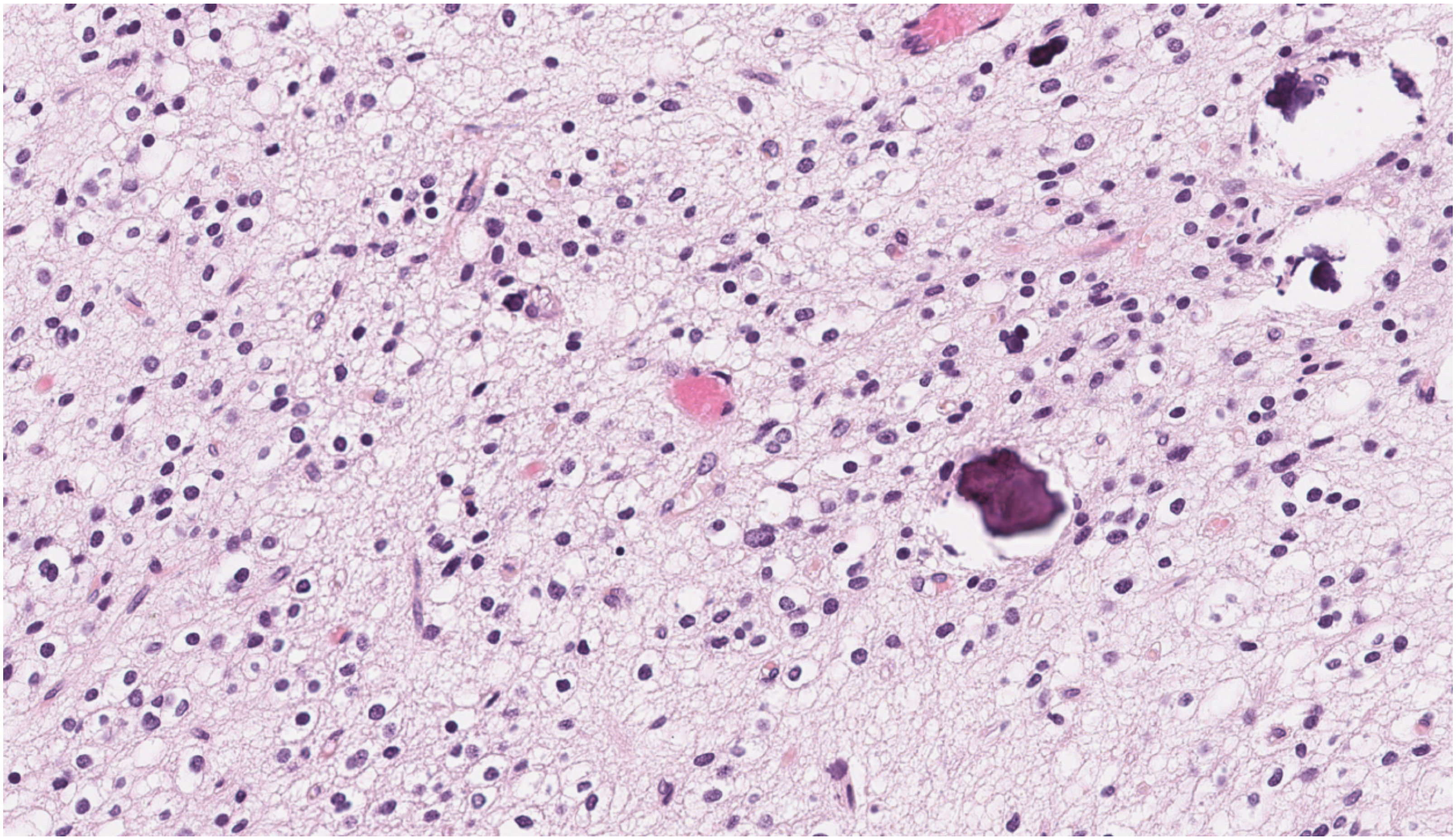

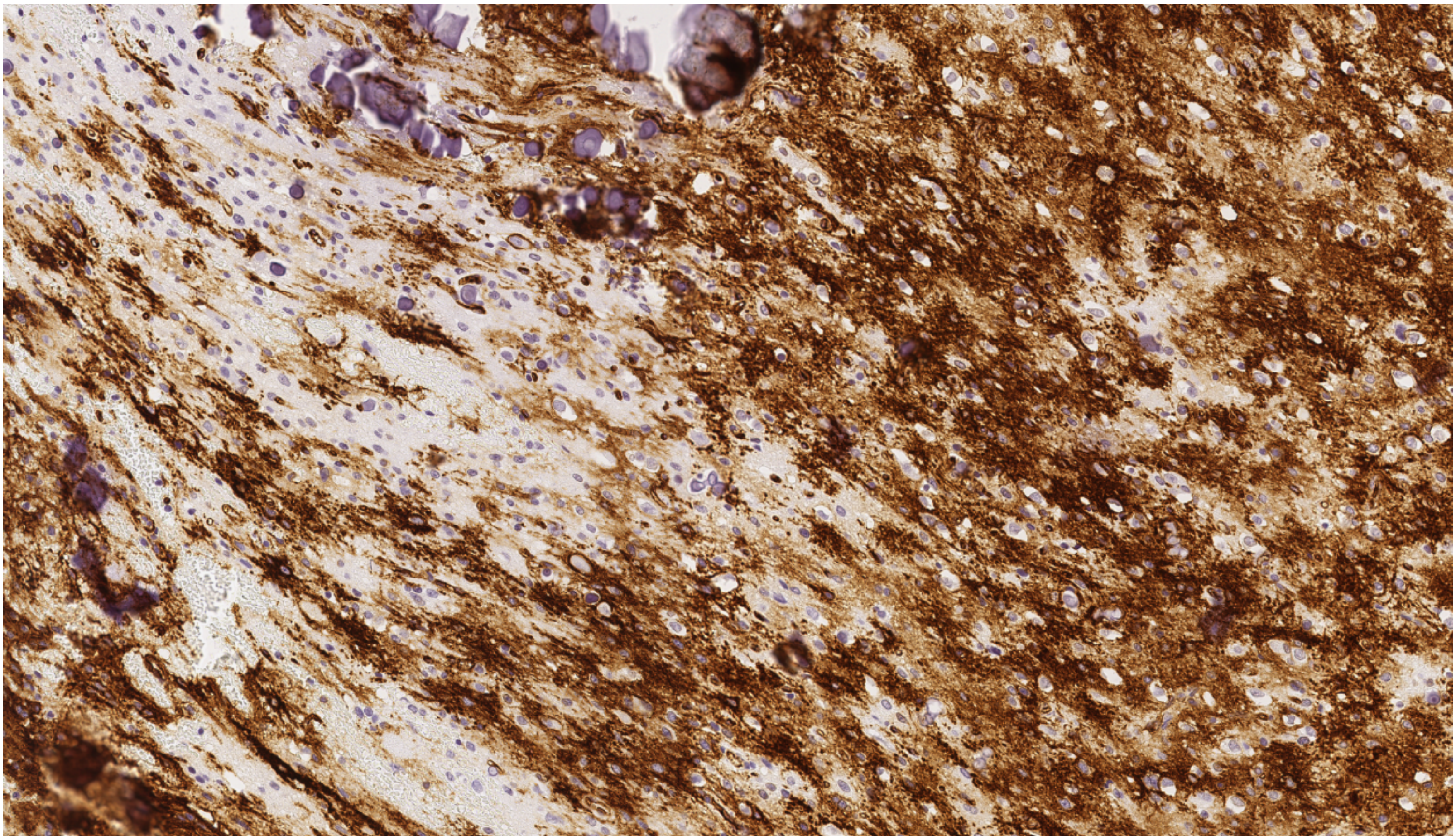

Patient #5, 7 y/o M, *FGFR2-KIAA1598* fusion, institutional histologic diagnosis = Ganglioglioma

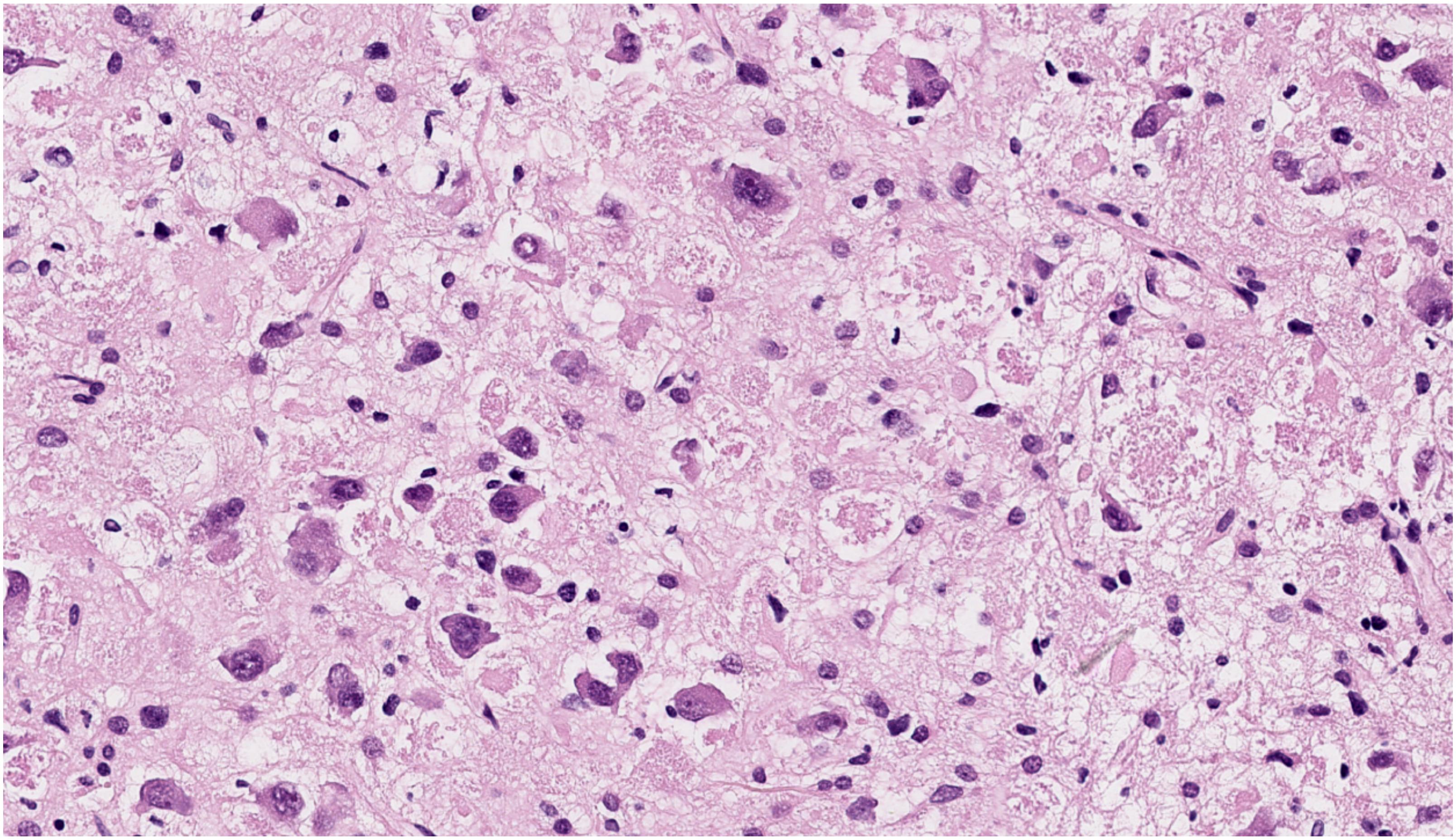

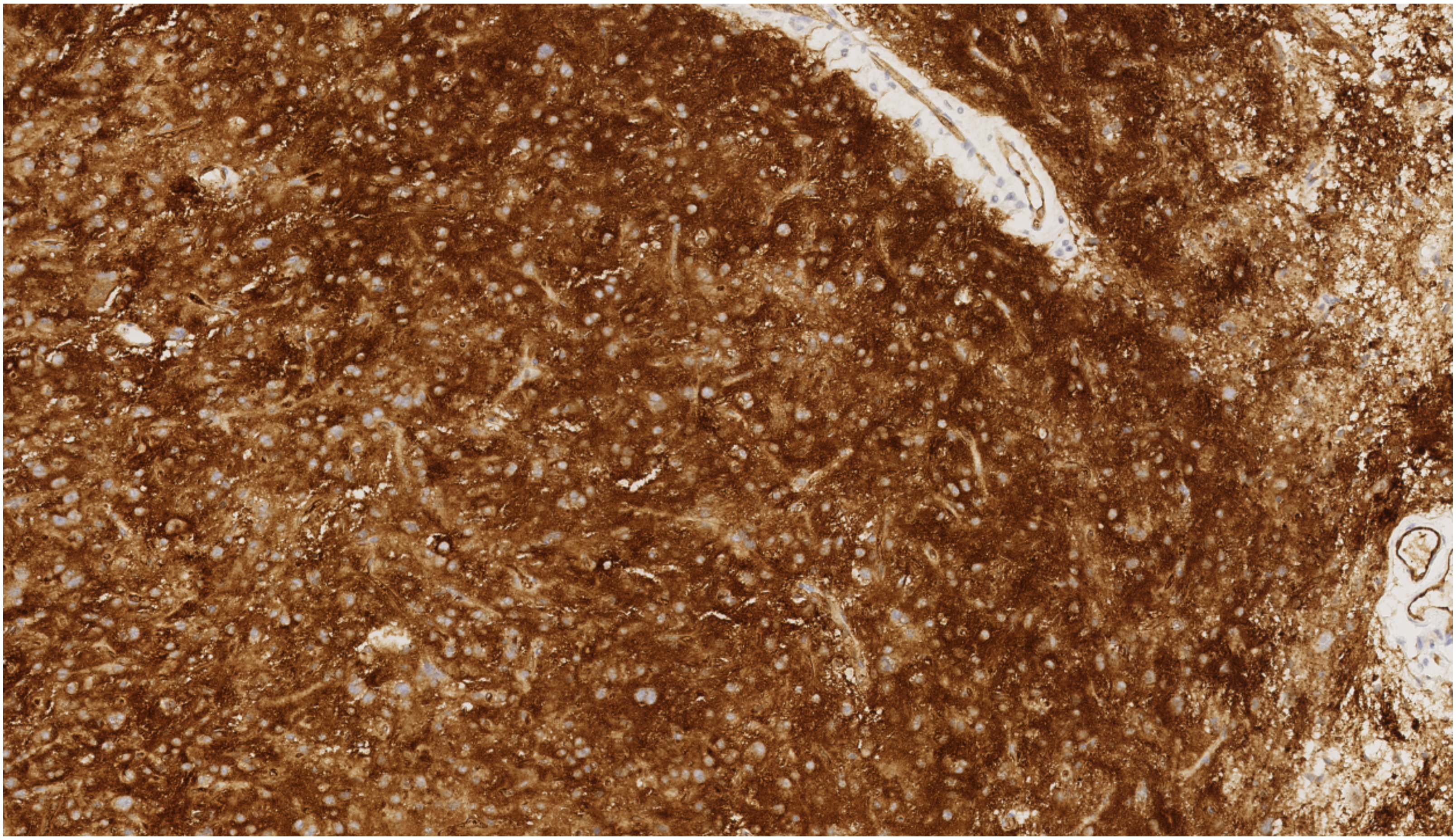

Patient #6, 35 y/o M, *FGFR2-INA* fusion, institutional histologic diagnosis = Ganglioglioma

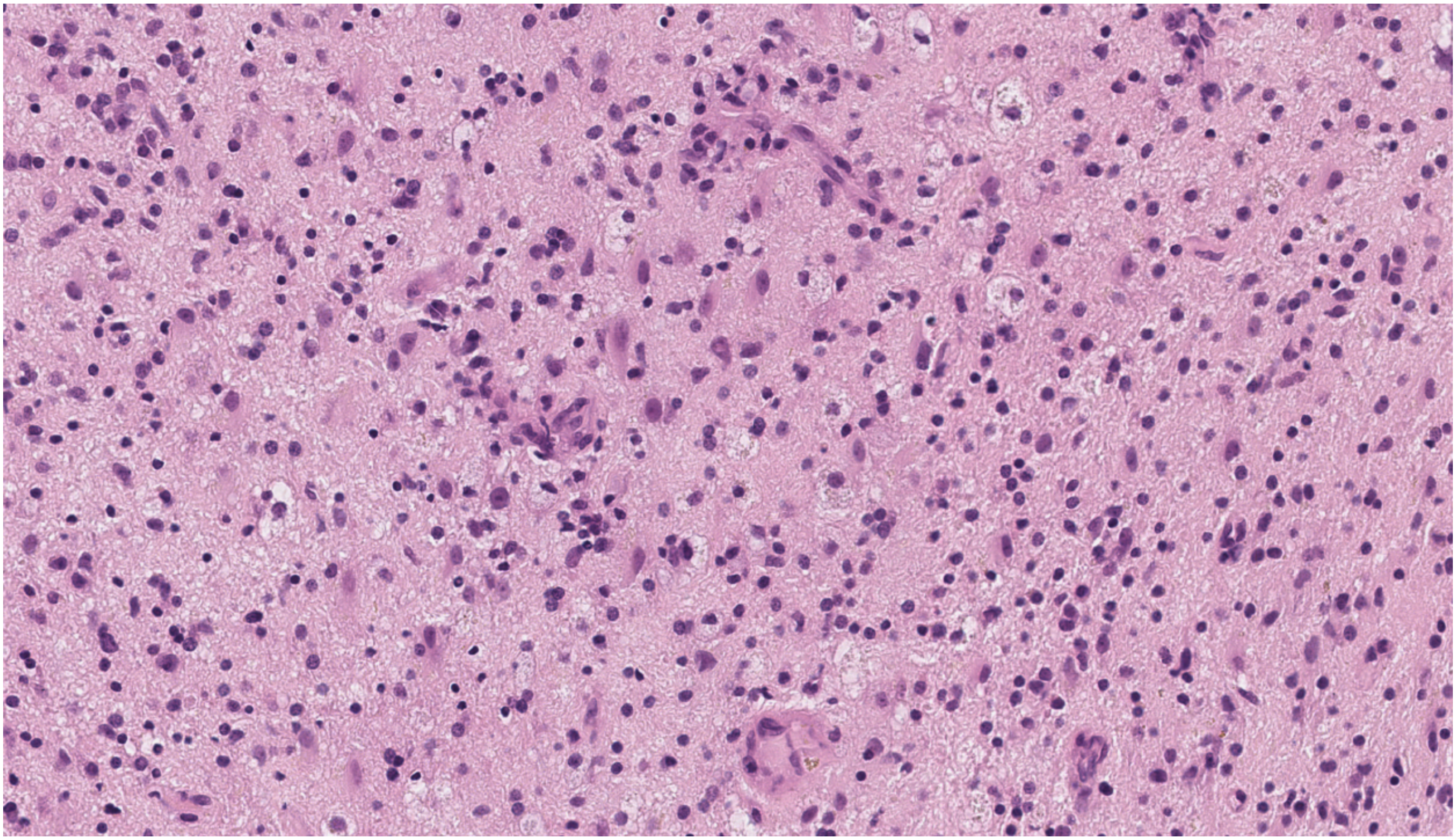

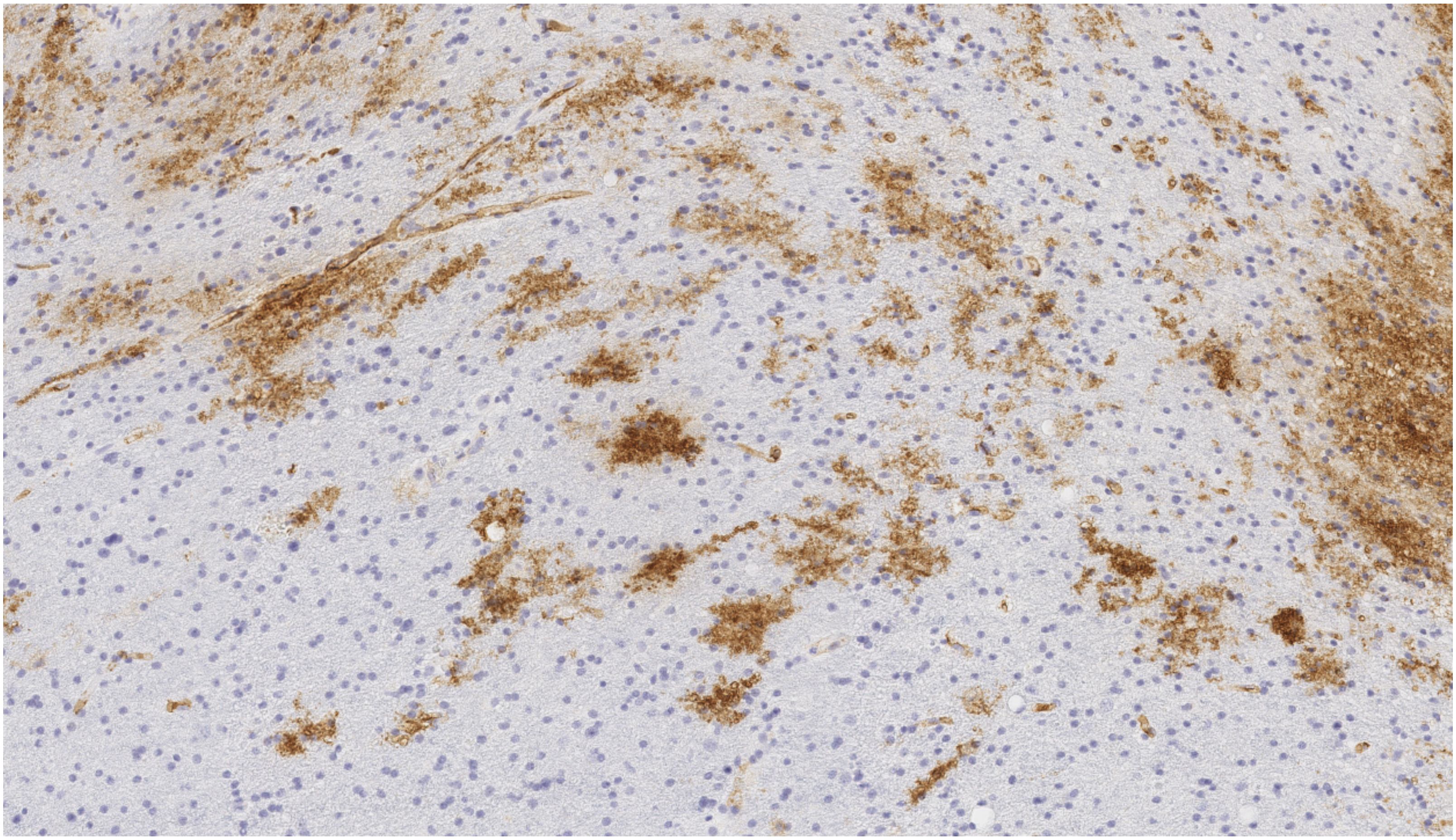

Patient #7, 38 y/o M, *FGFR2-INA* fusion, institutional histologic diagnosis = PLNTY

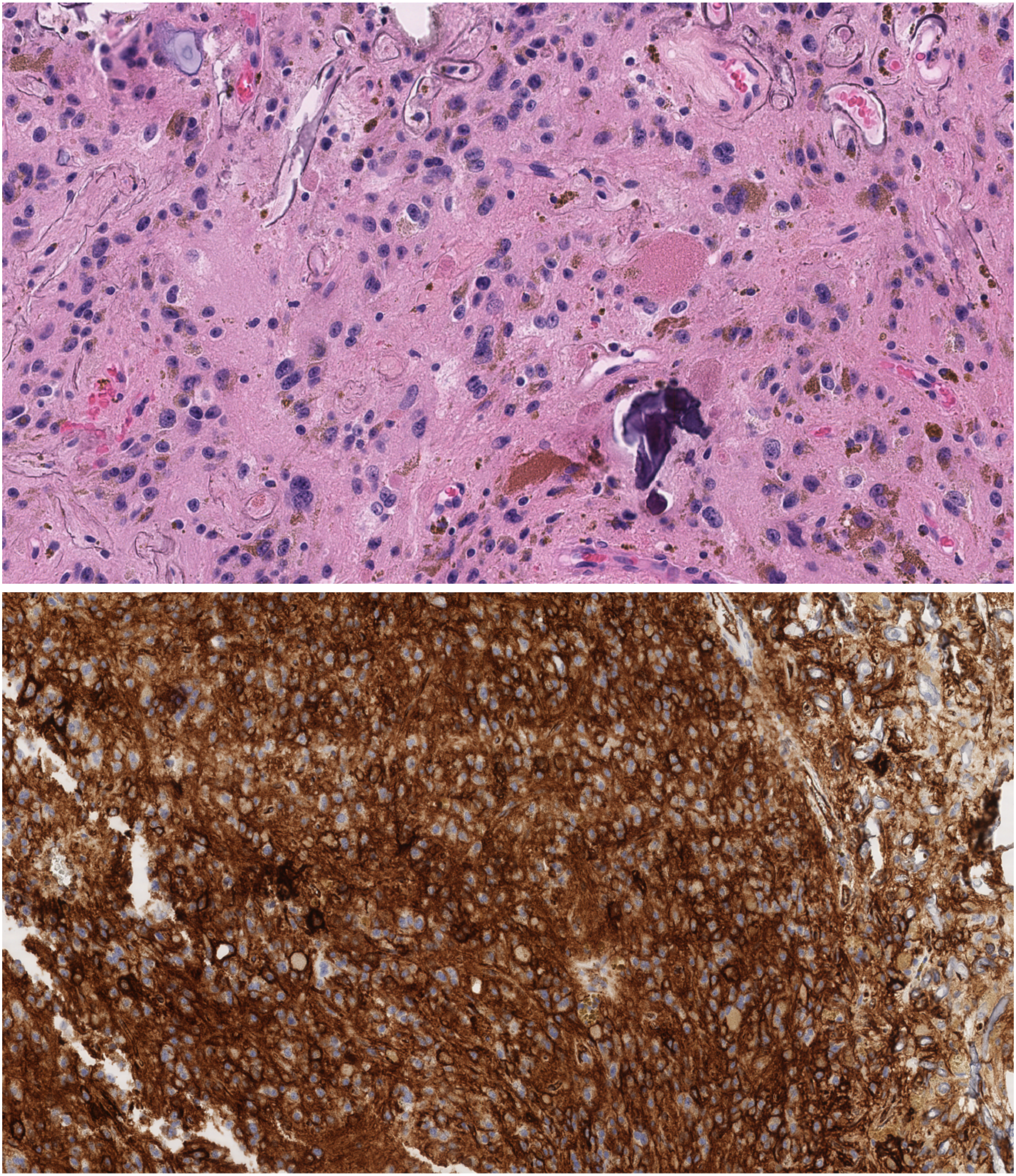

Patient #8, 6 y/o F, *FGFR2-OPTN* fusion, institutional histologic diagnosis = unclassifiable LGNET, favor DNT

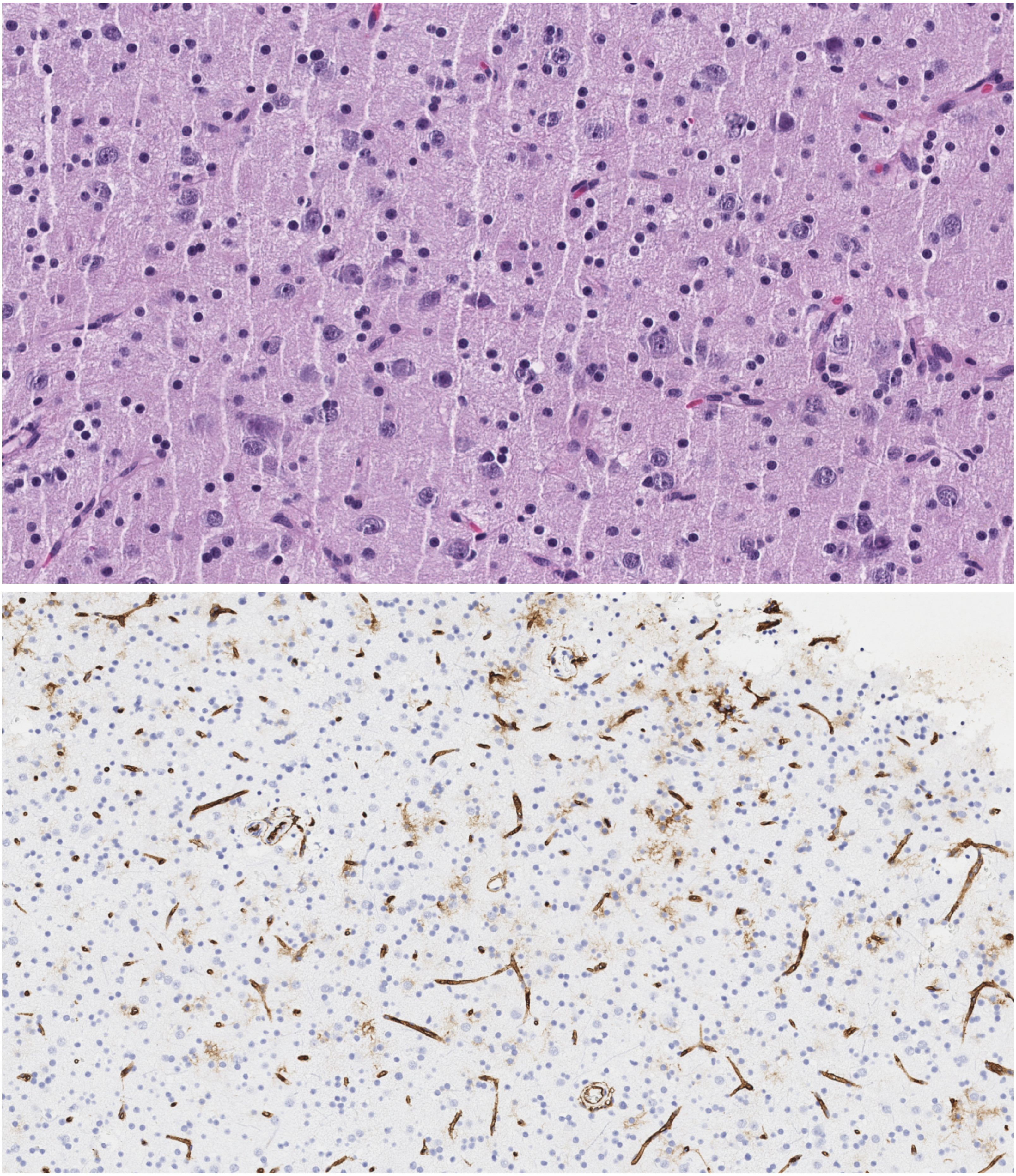

Patient #9, 11 y/o F, *FGFR2* rearrangement, institutional histologic diagnosis = PLNTY

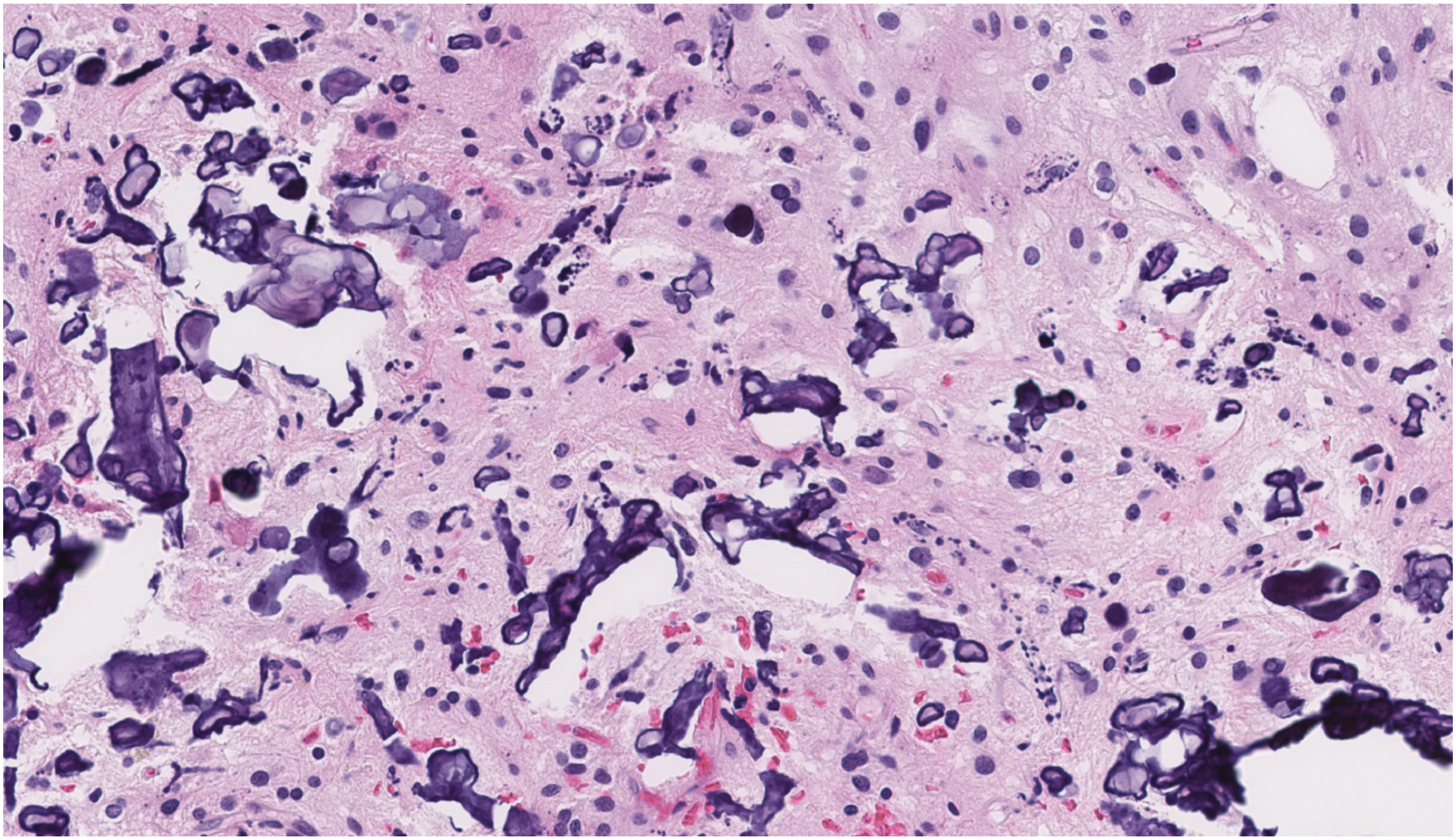

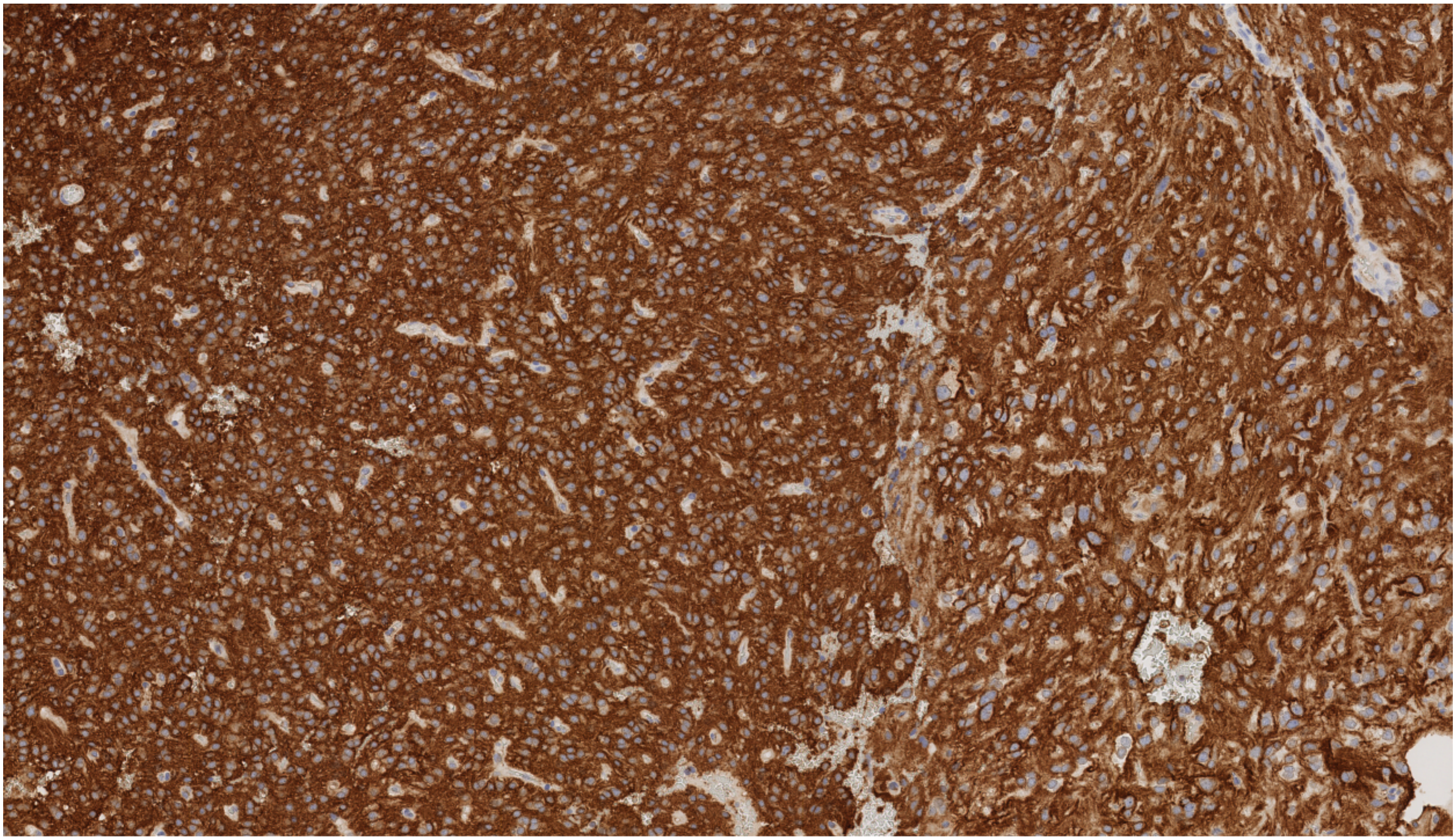

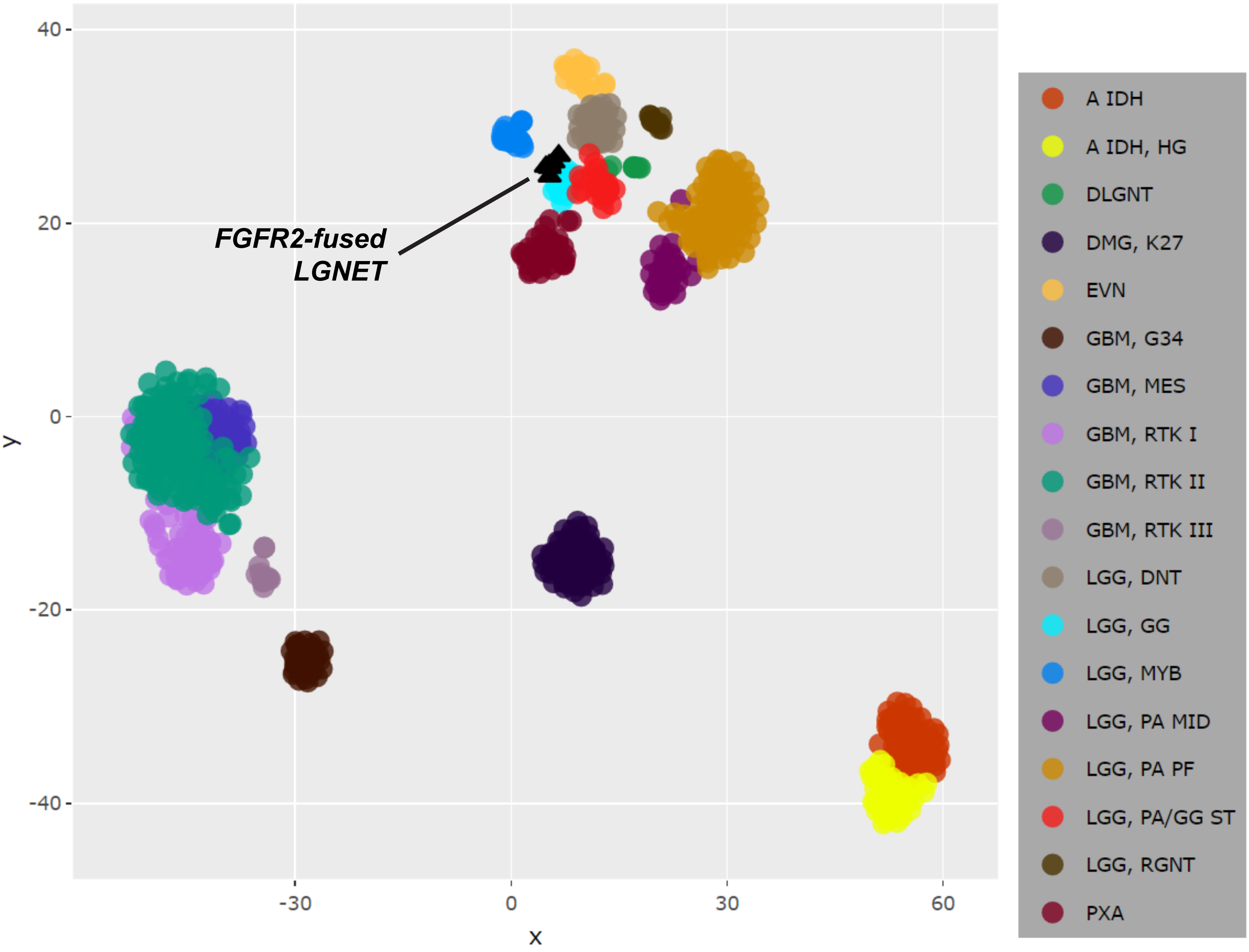


**Supplementary Figure 4.** Low-grade neuroepithelial tumors with *FGFR2* fusion resolve into a distinct epigenetic group. tSNE plot of genome-wide DNA methylation profiles from 8 *FGFR2*-fused LGNET alongside 865 reference tumors spanning 18 CNS tumor entities.

A IDH - astrocytoma, IDH-mutant; A IDH, HG - astrocytoma, IDH-mutant, high-grade; DLGNT - diffuse leptomeningeal glioneuronal tumor; DMG, K27 - diffuse midline glioma, H3 K27-mutant; EVN - extraventricular neurocytoma; GBM-G34 - diffuse hemispheric glioma, H3 G34-mutant; GBM, MES - glioblastoma, IDH-wildtype, mesenchymal subclass; GBM, RTK I - glioblastoma, IDH-wildtype, RTK1 subclass; GBM, RTK II - glioblastoma, IDH-wildtype, RTK2 subclass; GBM, RTK III - glioblastoma, IDH-wildtype, RTK3 subclass; LGG, DNT - dysembryoplastic neuroepithelial tumor; LGG, GG - ganglioglioma; LGG, MYB - pediatric-type diffuse low-grade glioma, MYB/MYBL1 fusion positive; LGG, PA MID - pilocytic astrocytoma, midline subclass; LGG, PA PF - pilocytic astrocytoma, posterior fossa subclass; LGG, PA/GG ST - pilocytic astrocytoma, supratentorial subclass; LGG, RGNT - rosette-forming glioneuronal tumor; PXA - pleomorphic xanthoastrocytoma

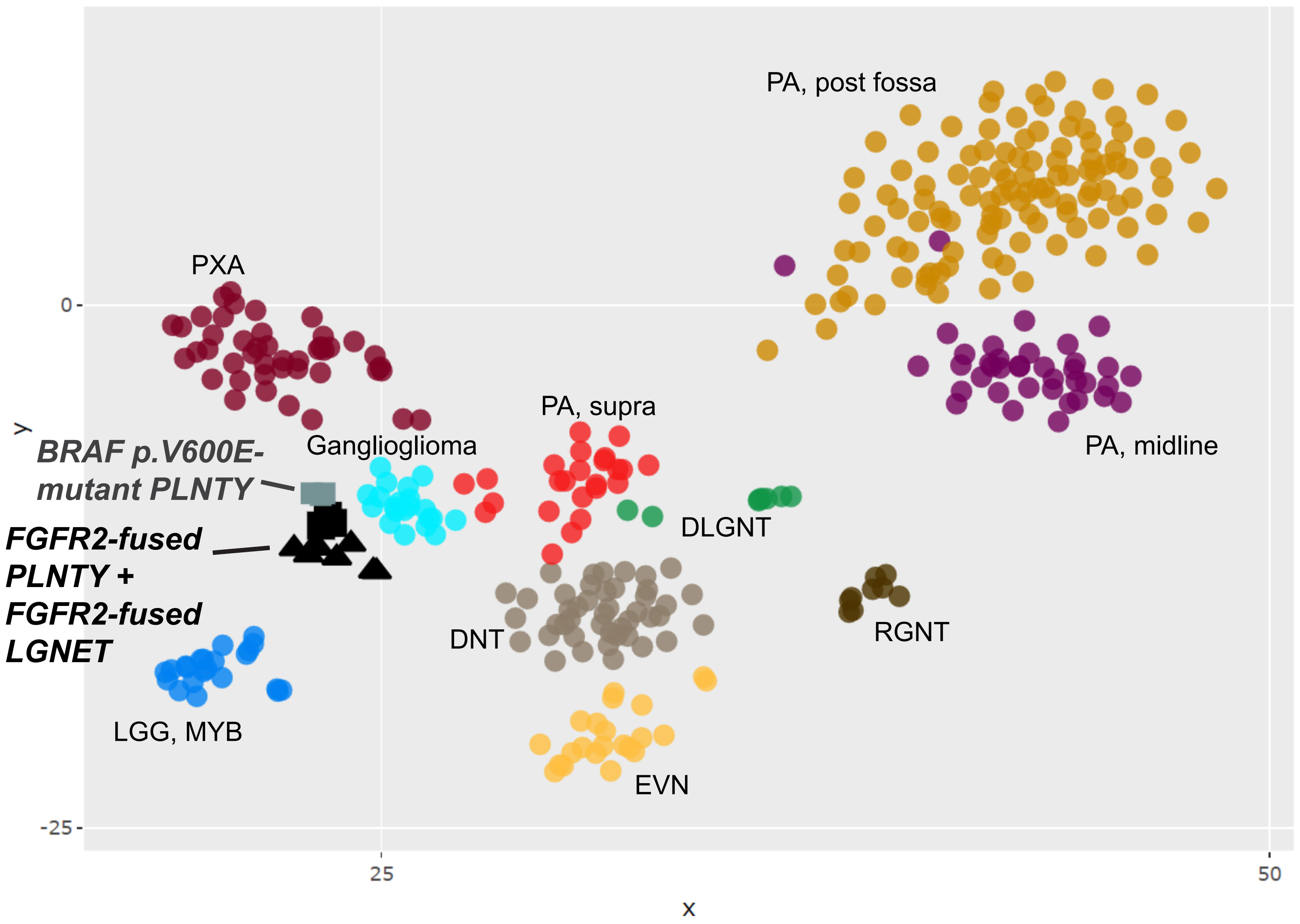


**Supplementary Figure 5.** tSNE plot of genome-wide DNA methylation profiles from the 8 *FGFR2*-fused LGNET (black triangles) alongside 3 histologically-defined PLNTY with *FGFR2* fusion (black squares) and 3 histologically-defined PLNTY with *BRAF* p.V600E mutation (gray squares) from Huse et al. [ref. 5], together with 346 reference CNS tumors spanning 10 LGNET entities.

DLGNT - diffuse leptomeningeal glioneuronal tumor; DNT - dysembryoplastic neuroepithelial tumor;
EVN, extraventricular neurocytoma; LGG, MYB - pediatric-type diffuse low-grade glioma, MYB/MYBL1 fusion positive;
PA, midline - pilocytic astrocytoma, midline subclass; PA, post fossa - pilocytic astrocytoma, posterior fossa subclass;
PA, supra - pilocytic astrocytoma, supratentorial subclass; PLNTY - polymorphous low-grade neuroepithelial tumor of the young; RGNT - rosette-forming glioneuronal tumor; PXA - pleomorphic xanthoastrocytoma

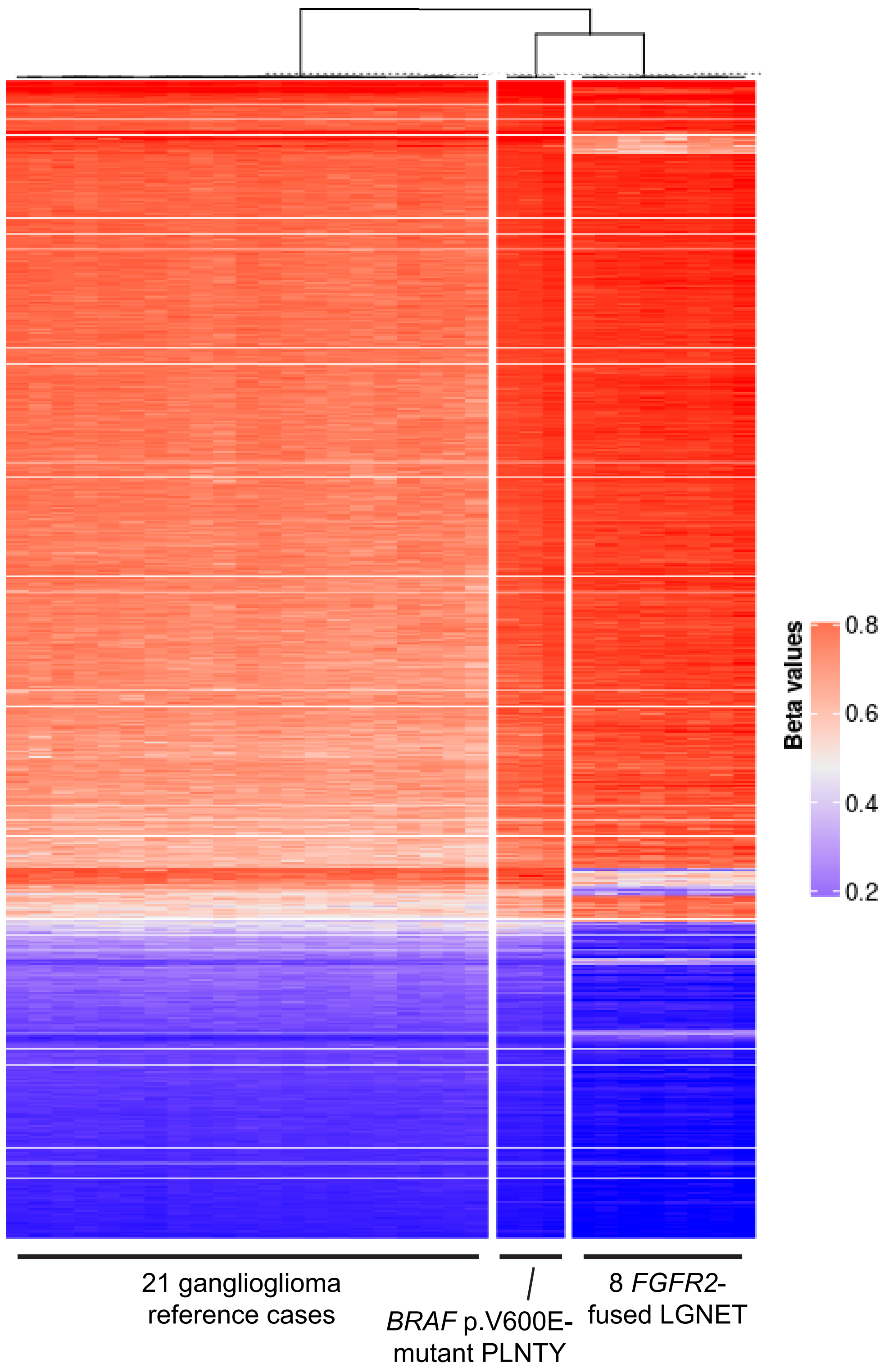


**Supplementary Figure 6.** Unsupervised hierarchical clustering of DNA methylation data from the 8 LGNET with *FGFR2* fusion alongside 21 reference ganglioglioma cases and 3 histologically-defined polymorphous low-grade neuroepithelial tumors of the young (PLNTY) with *BRAF* p.V600E mutation from Huse et al. [ref. 5], which demonstrates three distinct epigenetic clusters.
