# Supplementary material for: Low-grade glioneuronal tumors with FGFR2 fusion resolve into a single epigenetic group corresponding to ‘Polymorphous low-grade neuroepithelial tumor of the young’
Source: Acta Neuropathol. 2021 Jul 28;142(3):595–9. doi: 10.1007/s00401-021-02352-w (PMC8357689; doi:10.1007/s00401-021-02352-w)
Supplement: Supplementary file 3 — Online Resource 3. Supplementary Methods. (PDF 401 kb) [file 401_2021_2352_MOESM3_ESM.pdf]

Online Resource 3. Supplementary Methods.

Patient cohort and tumor samples

Nine patients with LGNET with *FGFR2* fusions or rearrangements identified by capture-based next-generation DNA sequencing were included in this study. All tumor specimens were fixed in 10% neutral-buffered formalin and embedded in paraffin. Tumor tissue was selectively scraped from unstained slides or punched from formalin-fixed, paraffin-embedded blocks using 2.0 mm disposable biopsy punches (Integra Miltex Instruments, cat# 33-31-P/25) to enrich for as high of tumor content as possible. Genomic DNA was extracted from this macrodissected formalin-fixed, paraffin-embedded tumor tissue using the QIAamp DNA FFPE Tissue Kit (Qiagen).

Targeted next-generation sequencing

Targeted next-generation sequencing was performed using the UCSF500 NGS Panel as previously described [Kline et al]. Capture-based next-generation DNA sequencing was performed using an assay that targets all coding exons of 479 cancer-related genes, select introns and upstream regulatory regions of 47 genes to enable detection of structural variants including gene fusions, and DNA segments at regular intervals along each chromosome to enable genome-wide copy number and zygosity analysis, with a total sequencing footprint of 2.8 Mb. Multiplex library preparation was performed using the KAPA Hyper Prep Kit (Roche) according to the manufacturer's specifications using 250 ng of sample DNA. Hybrid capture of pooled libraries was performed using a custom oligonucleotide library (Nimblegen SeqCap EZ Choice). Captured libraries were sequenced as paired-end 100 bp reads on an Illumina HiSeq 2500 instrument. Sequence reads were mapped to the reference human genome build GRCh37 (hg19) using the Burrows-Wheeler aligner (BWA). Recalibration and deduplication of reads was performed using the Genome Analysis Toolkit (GATK). Coverage and sequencing statistics were determined using Picard CalculateHsMetrics and Picard CollectInsertSizeMetrics. Single nucleotide variant and small insertion/deletion mutation calling was performed with FreeBayes, Unified Genotyper, and Pindel. Large insertion/deletion and structural alteration calling was performed with Delly. Variant annotation was performed with Annovar. Single nucleotide variants, insertions/deletions, and structural variants were visualized and verified using Integrative Genome Viewer. Genome-wide

copy number and zygosity analysis was performed by CNVkit and visualized using Nexus Copy Number (Biodiscovery).

DNA methylation profiling

Genomic DNA from 8 *FGFR2*-fused LGNET was bisulfite converted using the EZ DNA Methylation kit following the manufacturer's recommended protocol (Zymo Research). Bisulfite converted DNA was then amplified, fragmented, and hybridized to Infinium EPIC 850k Human DNA Methylation BeadChips following the manufacturer's recommended protocol (Illumina). Methylation data were preprocessed using the minfi package (v.1.30.0) in R Bioconductor (version 3.5.3) [Aryee et al]. The detection p-value for each sample was computed, and CpG sites with detection p values above 0.05 were discarded from the analysis. Additional quality control was performed by calculating the median log (base2) intensities for methylated and unmethylated signals for each array. All samples had unmethylated and methylated median intensity values above 10 that were used for analysis. Functional normalization with NOOB background correction and dye-bias normalization was performed [Fortin et al; Triche et al]. Probe filtering was performed after normalization. Specifically, probes located on sex chromosomes, containing nucleotide polymorphisms (dbSNP132 Common) within five base pairs of and including the targeted CpG site, or mapping to multiple sites on hg19 (allowing for one mismatch), as well as cross reactive probes were removed from analysis.

The DNA methylation profiles of the 8 tumors were assessed together with 865 reference tumors spanning 18 CNS tumor entities previously generated at DKFZ [Capper et al; Sievers et al]. These included 78 A IDH (astrocytoma, IDH-mutant), 46 A IDH-HG (astrocytoma, IDH-mutant, high-grade), 8 DLGNT (diffuse leptomeningeal glioneuronal tumor), 78 DMG-K27 (diffuse midline glioma, H3 K27M-mutant), 22 EVN (extraventricular neurocytoma), 41 GBM-G34 (diffuse hemispheric glioma, H3 G34-mutant), 56 GBM MES (glioblastoma, IDH-wildtype, mesenchymal subclass), 64 GBM RTK1 (glioblastoma, IDH-wildtype, RTK1 subclass), 143 GBM RTK2 (glioblastoma, IDH-wildtype, RTK2 subclass), 13 GBM RTK3 (glioblastoma, IDH-wildtype, RTK3 subclass), 44 DNT (dysembryoplastic neuroepithelial tumor), 21 GG (ganglioglioma), 22 LGG MYB (low-grade glioma, MYB/MYBL1 fusion positive), 38 PA MID (pilocytic astrocytoma, midline subclass), 114 PA PF (pilocytic astrocytoma, posterior fossa subclass), 24 PA ST (pilocytic astrocytoma, supratentorial subclass), 9 RGNT (rosette-forming

glioneuronal tumor), and 44 PXA (pleomorphic xanthoastrocytoma). Since the reference cohort contained methylation data generated using the Infinium Human Methylation 450k BeadChips, the approximately 450,000 overlapping CpG sites between the EPIC 850k and 450k BeadChips were used in the analysis. A beta value matrix with 389,282 CpG probes was used for all downstream analysis. Row-wise standard deviation was calculated for each probe across all samples, and the 32,502 most differentially methylated probes with standard deviation >0.216 were selected. Dimensionality reduction using t-distributed stochastic neighbor embedding (t-SNE) was performed by *Rtsne* (v.0.15) using the following analysis parameters: dims = 2, verbose=FALSE, max_iter = 3000, pca=F, theta=0, perplexity = 30, eta=200, num_threads=6. The tSNE plot was visualized with ggplot2 (v 3.2.0) [https://ggplot2.tidyverse.org/].

We performed unsupervised hierarchical clustering with the hclust function in Rstats (v3.6.0) to assess variation in DNA methylation patterns among the 8 *FGFR2*-fused LGNET and a reference cohort of 21 gangliogliomas from DKFZ [Capper et al]. The lmFit function from the Limma package (v.3.40.6) was applied on a log-transformed β-value matrix to identify the 20,000 most differentially methylated CpG probes across the two tumor cohorts. Then K-means clustering utilizing the Pearson distance matrix with complete linkage was used to determine the optimal number of clusters, through 500 re-sampling interactions of the dataset for K-means of 2, 3, 4, or 5. Visualization was performed using the R package ComplexHeatmap (v.2.0.0) [Gu et al]. Differentially methylated regions (DMRs) between the two tumor cohorts were identified using DMRcate (v1.20.0) [Peters et al], which ranks the most differentially methylated genomic regions using gaussian smoothing across adjacent CpG sites. For DMRcate, a model with coefficients using the following parameters and thresholds was created: lambda (genomic window length) = 1000 nucleotides, C (scaling factor) = 2, and probe false discovery rate (FDR) cutoff of less than 0.05. Results were ranked by Fisher's multiple comparison statistic and filtered for those DMRs with both FDR and Stouffer scores less than 0.001. DMRs were then annotated to the nearest gene transcriptional start sites, based on ENSEMBL genome annotations. Gene Ontology (GO) analysis of differentially methylated gene regions was performed using the gometh function in the missMethyl package [Phipson et al].

List of the 479 genes targeted for sequencing on the version of the UCSF500 NGS Panel used for tumor samples in this study. All coding exons were captured for sequencing from each of these genes, with those genes highlighted in red also having select intronic or upstream regulatory regions that were captured for sequencing to enable detection of structural variants.

UCSF 500 Version 2 Gene List

ABL1	ABL2	ACVR1	ACVR1B	AJUBA	AKT1	AKT2	AKT3	ALK	APC	APOBEC3G
AR	ARAF	ARFRP1	ARHGAP35	ARID1A	ARID1B	ARID2	ARID5B	ASH2L	ASXL1	ASXL2
ATF1	ATM	ATR	ATRX	AURKA	AURKB	AXIN1	AXIN2	AXL	BAP1	BARD1
BCL2	BCL2A1	BCL2L1	BCL2L12	BCL2L2	BCL6	BCOR	BCORL1	BLM	BRAF	BRCA1
BRCA2	BRD4	BRIP1	BTG1	BTK	CALR	CARD11	CBFB	CBL	CBLB	CCND1
CCND2	CCND3	CCNE1	CD79A	CD79B	CD274	CDC42	CDC73	CDH1	CDK12	CDK4
CDK6	CDK8	CDKN1A	CDKN1B	CDKN2A	CDKN2B	CDKN2C	CEBPA	CHD1	CHD2	CHD4
CHD5	CHEK1	CHEK2	CIC	CLDN18	CNOT3	COL1A1	COL2A1	CRCT1	CREB1	CREBBP
CRKL	CSF1R	CSF3R	CTCF	CTNNA1	CTNNB1	CUL3	CUX1	CYLD	CXCR4	DCC
DDIT3	DDR2	DDX3X	DDX41	DGKH	DICER1	DIS3	DNAJB1	DNMT3A	DOT1L	DUSP2
DUSP4	DUSP6	DYNC1I1	EBF1	EDNRB	EGFR	EGR1	EIF1AX	ELF3	EMSY (C11orf30)	EP300
EPCAM	EPHA2	EPHA3	EPHA5	EPHA7	EPHB1	EPOR	ERBB2	ERBB3	ERBB4	ERCC1
ERCC2	ERG	ERRFI1	ESPL1	ESR1	ESR2	ETS1	ETV6	EWSR1	EZH1	EZH2
FAM123B (WTX)	FAM46C	FANCA	FANCC	FANCE	FANCF	FANCG	FANCL	FAT1	FAT3	FBXW7
FGF10	FGF14	FGF19	FGF23	FGF3	FGF4	FGF6	FGFR1	FGFR2	FGFR3	FGFR4
FH	FLCN	FLT1	FLT3	FLT4	FOXA1	FOXL2	FOXO1	FOXP1	FRS2	FUBP1
FUS	FYN	GAB2	GATA1	GATA2	GATA3	GLI1	GLI2	GNA11	GNA13	GNAQ
GNAS	GPC3	GPR124 (ADGRA2)	GRIN2A	GRM3	GSK3B	H3F3A	H3F3B	HDAC4	HDAC9	HEY1
HGF	HIF1A	HIST1H3B	HMGA2	HNF1A	HOXB13	HRAS	HSPA2	HSPA5	HSP90AB1	ID3
IDH1	IDH2	IGF1R	IGF2	IGF2R	IKBKE	IKZF1	IKZF2	IKZF3	IL2RB	IL7R
INHBA	INPP4B	IPMK	IRF4	IRS2	JAK1	JAK2	JAK3	JAZF1	KAT6A (MYST3)	KDM5A
KDM5C	KDM6A	KDR	KEAP1	KIT	KLF4	KLHL6	KMT2A	KMT2B	KMT2D	KNSTRN
KRAS	LEF1	LIFR	LRP1B	LRRK2	LZTR1	MALAT1	MAML2	MAP2K1	MAP2K2	MAP2K4
MAP3K1	MAP3K2	MAP3K5	MAP3K7	MAP3K9	MAPK1	MCL1	MDM2	MDM4	MED12	MEF2B
MEN1	MET	MGA	MGMT	MITF	MLH1	MLH3	MPL	MRE11A	MSH2	MSH3
MSH6	MTOR	MUTYH	MYB	MYBL1	MYC	MYCL1	MYCN	MYD88	MYH9	NAV3
NBN	NCKAP5	NCOA2	NCOA3	NCOR1	NF1	NF2	NFE2L2	NFKBIA	NFKBIE	NIPBL
NKX2-1	NOTCH1	NOTCH3	NPM1	NRAS	NSD1	NSD2	NT5C2	NTRK1	NTRK2	NTRK3
NUP93	NUTM1	OR5L1	PAK1	PAK3	PALB2	PARK2	PAX3	PAX5	PAX7	PAX8
PBRM1	PDCD1LG2	PDGFB	PDGFRA	PDGFRB	PDK1	PHF6	PHOX2B	PIK3CA	PIK3CG	PIK3R1
PIK3R2	PLAG1	PLCB4	PMS1	POLD1	POLE	POLQ	POT1	POU3F2	PPM1D	PPP2R1A
PPP6C	PRDM1	PREX2	PRKACA	PRKAG2	PRKAR1A	PRKCA	PRKCH	PRKDC	PTCH1	PTCH2
PTEN	PTK2B	PTPN1	PTPN11	PTPRB	PTPRD	PTPRK	PTPRT	RAC1	RAD21	RAD50
RAD51	RAD51C	RAD51D	RAF1	RARA	RASA1	RASA2	RB1	RBM10	REL	RELA
RET	RHEB	RHOA	RICTOR	RIT1	RNF43	ROBO1	ROS1	RPL10	RPTOR	RRAGC
RRAS	RRAS2	RSPO2	RSPO3	RUNX1	RUNX1T1	SDHB	SDHD	SETBP1	SETD2	SF3B1
SH2B3	SHH	SIN3A	SLIT2	SLITRK6	SMAD2	SMAD3	SMAD4	SMARCA2	SMARCA4	SMARCB1
SMC1A	SMC3	SMO	SNCAIP	SOCS1	SOS1	SOS2	SOX9	SOX10	SOX2	SPEN
SPOP	SPRED1	SPRY1	SPRY2	SPRY4	SPTA1	SRC	SRSF2	SS18	STAG2	STAT3
STAT4	STAT6	STK11	SUFU	SYK	SYNE1	TADA1	TBX3	TCEB1	TCF7L2	TERT
TET2	TFE3	TFEB	TGFBR2	TLR4	TNFAIP3	TNFRSF14	TOP1	TOP2A	TMPRSS2	TP53
TRAF3	TRAF7	TRIM28	TSC1	TSC2	TSHR	TSHZ2	TSHZ3	TSLP	TTYH1	TYK2
U2AF1	USP7	VEGFA	VHL	WISP3	WRN	WT1	XBP1	XPO1	YAP1	YWHAE
ZBTB20	ZFHX3	ZMYM3	ZNF217	ZNF703	ZNFHX4	ZRSR2				

Gupta R, et al. Low-grade glioneuronal tumors with *FGFR2* fusion resolve into a single epigenetic group corresponding to 'Polymorphous low-grade neuroepithelial tumor of the young'

References for Supplementary Methods

Aryee MJ, Jaffe AE, Corrada-Bravo H et al (2014) Minfi: a flexible and comprehensive Bioconductor package for the analysis of Infinium DNA methylation microarrays. Bioinformatics 30:1363-1369

Capper D, Jones DTW, Sill M et al (2018) DNA methylation-based classification of central nervous system tumours. Nature 555:469-474

Fortin JP, Labbe A, Lemire M et al (2014) Functional normalization of 450k methylation array data improves replication in large cancer studies. Genome Biol 15:503

Gu Z, Eils R, Schlesner M (2016) Complex heatmaps reveal patterns and correlations in multidimensional genomic data. Bioinformatics 32:2847-2849

Kline CN, Joseph NM, Grenert JP et al (2017) Targeted next-generation sequencing of pediatric neuro-oncology patients improves diagnosis, identifies pathogenic germline mutations, and directs targeted therapy. Neuro Oncol 19:699-709

Peters TJ, Buckley MJ, Statham AL et al (2015) De novo identification of differentially methylated regions in the human genome. Epigenetics Chromatin 8:6.

Phipson B, Maksimovic J, Oshlack A (2016) missMethyl: an R package for analyzing data from Illumina's HumanMethylation450 platform. Bioinformatics 32:286-288

Sievers P, Stichel D, Schrimpf D et al (2018) FGFR1:TACC1 fusion is a frequent event in molecularly defined extraventricular neurocytoma. Acta Neuropathol 136:293-302.

Triche TJ, Weisenberger DJ, Van Den Berg D, Laird PW, Siegmund KD (2013) Low-level processing of Illumina Infinium DNA Methylation BeadArrays. Nucleic Acids Res 41:e90
